# Photochemical Generation of Allenes from Phenanthrene-Based Methylenecyclobutanes

**DOI:** 10.1021/acs.joc.5c00781

**Published:** 2025-06-09

**Authors:** Zhazira Koldasbay, Alexander D. Roth, Dasan M. Thamattoor

**Affiliations:** Department of Chemistry, 8439 Colby College, Waterville, Maine 04901, United States

## Abstract

Photofragmentation of substituted 1-methylene-1,2,2a,10b-tetrahydrocyclobuta­[*l*]­phenanthrenes, under ambient conditions in solution, produces the corresponding allenes with the concomitant formation of phenanthrene. These phenanthrene-based precursors offer a new and general photochemical route to allenes including, potentially, strained cyclic allenes.

## Graphical abstract

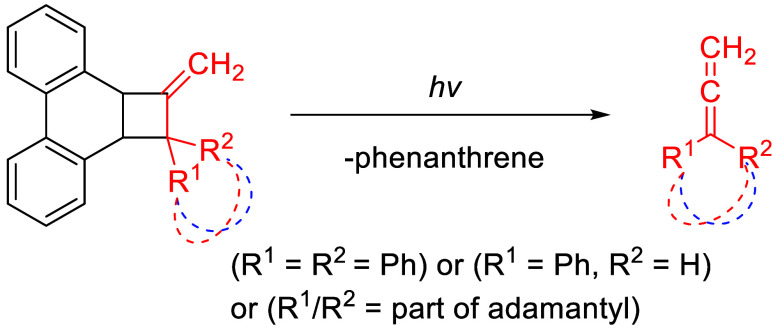

Allenes, characterized by their linear arrangement of two orthogonal π bonds connected via a common sp-hybridized central carbon atom, represent an important class of compounds in organic chemistry.^1,2^ Their unique structure and reactivity, and an architecture that is capable of conferring axial chirality, make them valuable synthons for the construction of chiral molecules,^3^ complex natural products,^4,5^ and advanced functional materials like shape-persistent macrocycles, foldamers, and chiral chromophores.^6^ It is estimated that there are over 150 natural products and pharmaceuticals that feature the allene moiety.^7^ Consequently, the development of efficient and versatile methods for synthesizing allenes is of considerable interest to the broader chemistry community.

Although several synthetic routes to allenes have been developed over the years,^1,8–15^ metal-free photochemical methods are sparse and limited to a few isolated examples.^1,16^ In this regard, it is of relevance to note a study by Tippmann and Curtis who reported that cyclobutanone **1** undergoes photofragmentation into phenanthrene and diphenylketene (**2**) upon laser flash photolysis at 266 nm (Scheme 1a).^17^ Our laboratory has recently disclosed that the steady state photolysis of **1** and related cyclobutanones, using a Xe­(Hg) lamp, can be a viable and generally applicable photochemical approach to ketenes.^18^ Building on our previous work, we herein describe the conversion of **1** and two related cyclobutanones into the corresponding methylenecyclobutanes **3**, and their subsequent photolysis to give allenes **4** by an analogous fragmentation of the four-membered ring (Scheme 1b). This approach holds promise as a broadly applicable photochemical route to a variety of acyclic allenes and could potentially be deployed to prepare strained cyclic allenes as well.

**Scheme 1:**
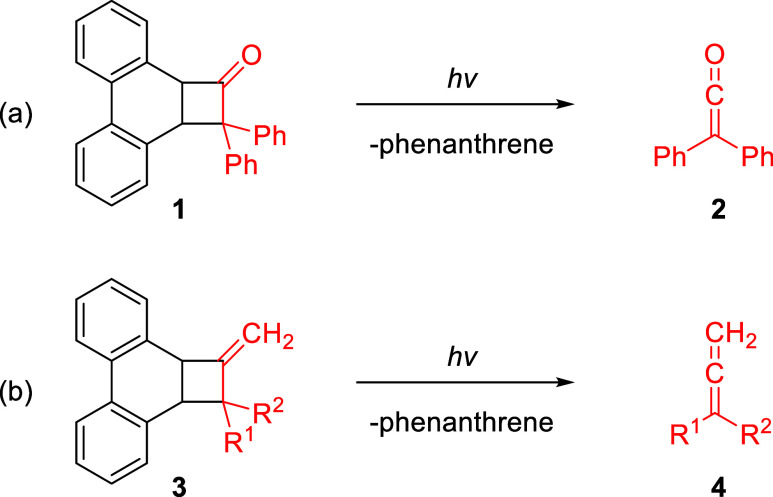
Photochemical Generation of (a) Ketene and (b) Allene from Phenanthrene-Based Cyclobutanes

## Synthesis of Phenanthrene-Based Methylenecyclobutane Precursors to Allenes

The cyclobutanones **1**, **6**, and **8**, all of which were synthesized by our recently published procedures,^18^ were converted in a single step to the corresponding allene precursors **5**, **7**, and **9** respectively by use of the Tebbe reagent (Scheme 2). The crystal structures of **5**, **7**, and **9** are shown in Figure 1.

**Figure 1:**
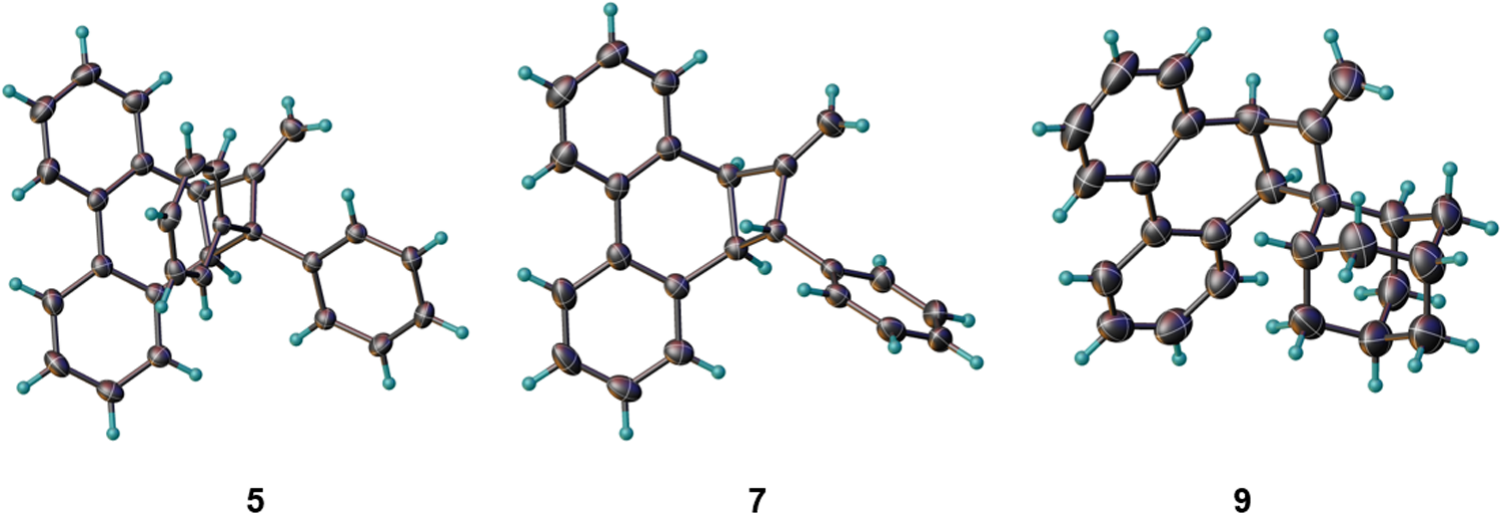
Single-crystal X-ray structures of **5**, **7**, and **9**. Thermal ellipsoids are shown at the 50% probability level.

**Scheme 2:**
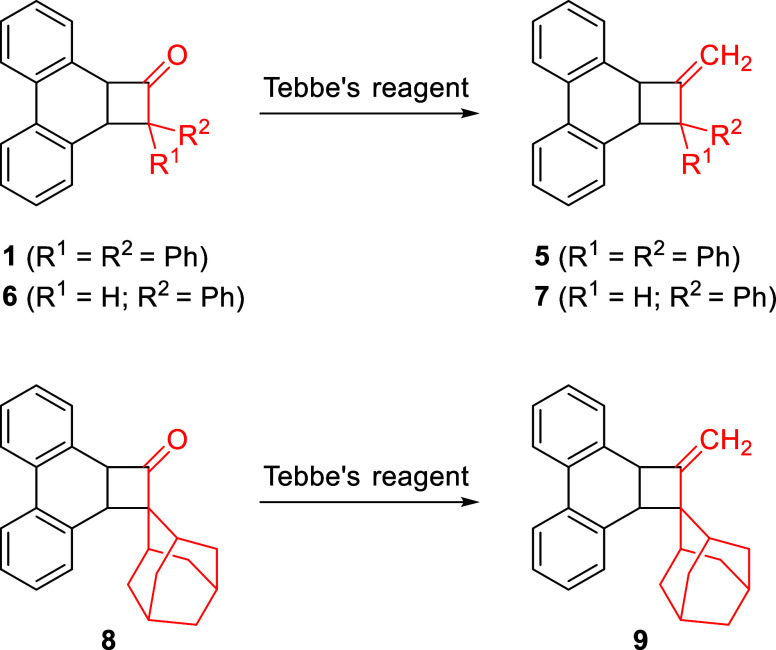
Synthesis of **5**, **7**, and **9**, Photochemical Precursors to Allenes by Tebbe Olefination

## Photochemical Experiments

Precursors 5, **7**, and **9** were dissolved in C_6_D_6_ and photolyzed at ambient temperature with a Xe–Hg lamp at 280–400 nm in a quartz NMR tube. The progress of the photolysis was monitored by ^1^H NMR spectroscopy. Photolysis was complete within an hour and the corresponding allenes **10**, **11**, and **12** were formed in yields of 56, 74, and 80% respectively as measured using *m*-xylene as an internal standard (Scheme 3). The identities of the allenes were confirmed by comparison to authentic samples that were synthesized independently.

**Scheme 3:**
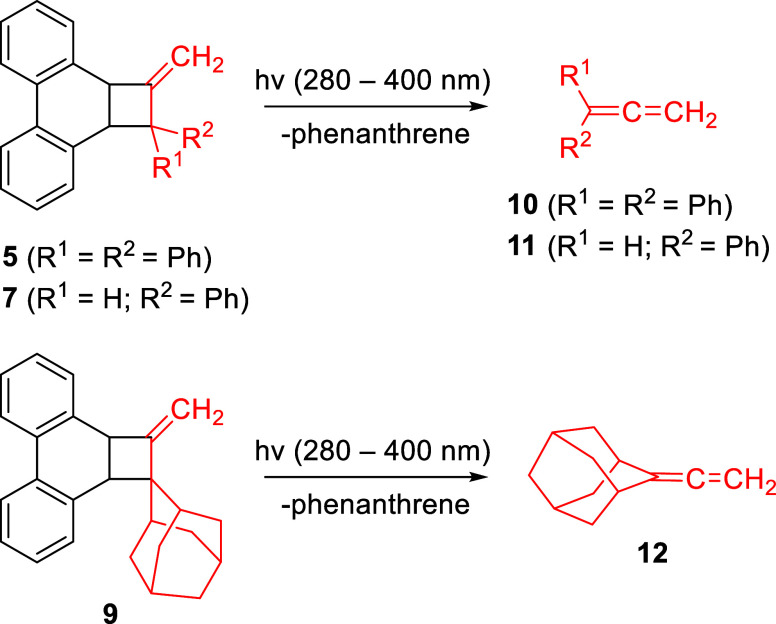
Photochemical Generation of Allenes **10**, **11**, and **12** from Precursors **5**, **7**, and **9**, Respectively

In conclusion, we report herein a new photochemical route to the generation of allenes from the fragmentation of phenanthrene-based methylenecyclobutanes. As a further extension of this method, one could envision the generation of chiral allenes from appropriately substituted precursors. In addition, precursors such as **13** could potentially serve as sources to strained cyclic allenes **14** (Scheme 4). Availability of precursors such as those described herein will also permit the investigation of allenes by methods such as laser flash photolysis and matrix isolation spectroscopy. Research along these lines is currently underway in our laboratory.

**Scheme 4:**
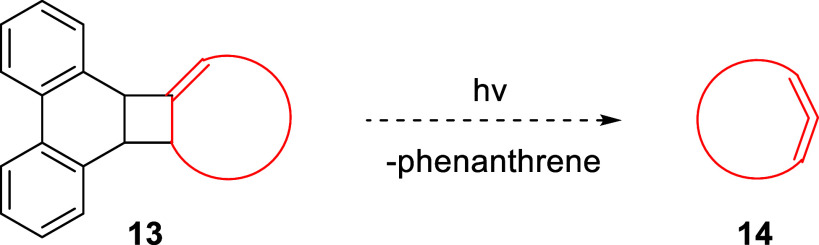
Potential Formation of Cyclic Allenes from Phenanthrene-Based Precursors

## Experimental Section

### General Information

Tetrahydrofuran was degassed by purging with nitrogen and dried by passage through two activated alumina columns (2 ft × 4 in). All other solvents and reagents were used as obtained from commercial sources. Synthetic reactions were carried out under an argon atmosphere in oven-dried glassware. Cyclobutanones **1**, **6**, and **8** were prepared according to literature procedures.^18^ Literature procedures were also used to synthesize authentic samples of diphenylallene (**10**),^19^ phenylallene (**11**),^20,21^ and adamantylideneallene (**12**).^22,23^

Medium pressure flash chromatography was performed on an automated system on prepacked silica gel columns (70–230 mesh) using hexanes as eluent. NMR spectra were recorded at 500 MHz for proton (^1^H) and 126 MHz for proton-decoupled carbon ^13^C­{^1^H} using CDCl_3_. The chemical shifts are reported in δ ppm with reference to the signal of tetramethylsilane set to 0 ppm. Infrared spectra (resolution 0.4 cm^–1^) were acquired with an Fourier transform infrared (FTIR) instrument equipped with an attenuated total reflectance (ATR) accessory and were processed with SpectraGryph.^24^ Gas chromatography–mass spectrometry (GC/MS) data were obtained with a capillary gas chromatograph interfaced with a quadrupole, triple-axis mass selective detector operating in the electron impact (EI) mode. High resolution mass spectra were obtained by electrospray ionization (ESI) on a time-of-flight mass spectrometer (TOF-MS). Melting points are uncorrected. UV/vis absorption spectra were recorded at room temperature on an Agilent Cary 60 UV/vis spectrophotometer (200–800 nm scan range with automated baseline correction) in a 1 cm quartz cuvette in acetonitrile.

Photolysis experiments were conducted with a Newport 200 W Xe­(Hg) arc lamp (model # 6290; horizontal intensity 600 cd) with a Newport 280–400 dichroic mirror (model # 66245) fitted in a Newport 67005 Housing with a Newport 69907 Universal Arc Lamp Power Supply. All photolysis reactions were conducted at ambient temperature in benzene-*d6* in 5 mm quartz NMR tubes positioned 30 cm away from the light source.

A Bruker D8 Quest Eco diffractometer equipped with a graphite monochromated Mo Kα radiation (λ = 0.71073 Å) and PHOTON 50 CMOS (**c**omplementary **m**etal-**o**xide **s**emiconductor) detector was used to collect X-ray diffraction data at 173 K with the Bruker Apex 4 suite of programs.^25^ Frames were integrated with a narrow-frame algorithm using the Bruker data reduction software package SAINT^26^ and absorption effects were corrected with the multiscan method (SADABS).^27^ The Olex2 suite of programs^28^ was used to process data along with the Bruker SHELXTL software package^29,30^ that was used to perform structure solution by direct methods, and refinement by full-matrix least-squares on F^2^. All nonhydrogen atoms were refined anisotropically with suggested weighting factors and the hydrogens were calculated on a riding model. All cif files were validated with the checkCIF/Platon facility of IUCr that was implemented through Olex2.^28^ Crystals suitable for X-ray diffraction were grown by slow diffusion of pentane into a solution of the compound in dichloromethane.

#### 2-Methylene-1,1-diphenyl-1,2,2a,10b-tetrahydrocyclobuta­[*l*]­phenanthrene (**5**)

To a solution of **1** (200 mg, 0.537 mmol) in THF (15.0 mL) was added Tebbe’s reagent (2.16 mL, 0.500 M in toluene, 1.08 mmol). After stirring at room temperature for 2 h, the reaction was then quenched with aqueous solution of NaOH (10.8 mL, 0.100 M), the aqueous layer was extracted with CH_2_Cl_2_ (3 × 10.0 mL), and the organic layer was then washed with H_2_O (2 × 10.0 mL), brine (3 × 10.0 mL), and the solid obtained after rotary evaporation was isolated as a white crystalline solid using flash-column chromatography with hexanes as eluent. The final yield was 136 mg (69%); mp: 110.8–113.1 °C. ^1^H NMR (500 MHz, CDCl_3_) δ 7.70 (dd, *J* = 8.1, 1.2 Hz, 1H), 7.64 (dd, *J* = 7.8, 1.3 Hz, 1H), 7.58–7.53 (m, 2H), 7.45–7.39 (m, 2H), 7.33 (d, *J* = 1.4 Hz, 2H), 7.25–7.17 (m, 2H), 7.12 (td, *J* = 7.6, 1.5 Hz, 1H), 6.97 (td, *J* = 7.4, 1.2 Hz, 1H), 6.86 (ddd, *J* = 7.8, 6.3, 1.7 Hz, 2H), 6.81–6.75 (m, 2H), 6.63–6.57 (m, 2H), 5.31 (dd, *J* = 2.1, 1.0 Hz, 1H), 5.07 (dd, *J* = 2.6, 0.9 Hz, 1H), 4.85 (d, *J* = 9.8 Hz, 1H), 4.41 (dt, *J* = 9.8, 2.4 Hz, 1H). ^13^C­{^1^H} NMR (126 MHz, CDCl_3_) δ 159.1, 145.8, 141.4, 133.2, 132.9, 131.9, 130.9, 129.6, 128.6, 128.3, 127.9, 127.8, 127.2, 126.9, 126.9, 126.5, 126.4, 125.6, 122.9, 122.8, 108.7, 45.3, 42.5. FTIR: ν=3055, 2918, 2851, 1671, 1596, 1489, 1444 cm^–1^. HRMS (ESI-TOF) *m*/*z* [M]^+^ calcd. for C_29_H_22_ 370.1722, found 370.1709. UV/vis (acetonitrile, *c* = 3.00 × 10^–5^ M, 1 cm path length): λ_max_ = 275 nm (ε = 8.80 × 10^3^ L mol^–1^ cm^–1^).

#### Methylene-2-phenyl-1,2,2a,10b-tetrahydrocyclobuta­[*l*]­phenanthrene (**7**)

To a stirred solution of compound **6** (50.0 mg, 0.169 mmol) in THF (10.0 mL) under argon was added Tebbe’s reagent (0.680 mL, 0.500 M in toluene, 0.340 mmol). The mixture was stirred at room temperature for 2 h, then quenched with aqueous solution of NaOH (3.40 mL, 0.100 M). The aqueous phase was extracted with CH_2_Cl_2_ (3 × 10.0 mL), the combined organic layers were washed with brine (10.0 mL), dried over Na_2_SO_4_, filtered, and concentrated under reduced pressure. Purification by silica-gel flash chromatography (hexanes) afforded the target product as a white crystalline solid (16.0 mg, 32%); mp: 87–91 °C. ^1^H NMR (500 MHz, CDCl_3_) δ: 7.97–7.91 (m, 2H), 7.39–7.26 (m, 9H), 7.17 (td, *J* = 7.4, 1.2 Hz, 1H), 7.00–6.96 (m, 1H), 5.23 (ddd, *J* = 2.6, 1.8, 0.7 Hz, 1H), 4.80 (td, *J* = 2.4, 0.7 Hz, 1H), 4.42 (dd, *J* = 9.3, 2.5 Hz, 1H), 4.22 (dq, *J* = 8.2, 2.8 Hz, 1H), 3.82–3.75 (m, 1H). ^13^C­{^1^H} NMR (126 MHz, CDCl3) δ: 155.7, 140.7, 135.4, 135.0, 131.4, 131.3, 129.4, 128.7, 128.6, 128.1, 127.9, 127.7, 127.3, 127.1, 126.7, 123.4, 123.2, 105.7, 58.8, 43.3, 42.9. FTIR (ATR): 3056, 3024, 2925, 2865, 1673, 1600, 1485, 1448, 1401 cm^–1^. HRMS (ESI-TOF) *m*/*z* [M]^+^ calcd. for C_23_H_18_ 294.1409, found 294.1426. UV/vis (acetonitrile, *c* = 3.50 × 10^–5^ M, 1 cm path length): λ_max_ = 275 nm (ε = 8.57 × 10^3^ L mol^–1^ cm^–1^).

#### 2′-Methylene-2a′,10b′-dihydro-2′*H*-spiro­[adamantane-2,1′-cyclobuta­[*l*]­phenanthrene] (**9**)

To a stirred solution of compound **8** (200 mg, 0.588 mmol) in THF (25.0 mL) under argon was added Tebbe’s reagent (2.30 mL, 0.500 M in toluene, 1.15 mmol), where it was allowed to stir for 1.5 h at room temperature. The reaction was quenched by addition of 0.100 M NaOH (12.0 mL), the aqueous layer was extracted with CH_2_Cl_2_ (3 × 20.0 mL) and the organic layer was washed with brine (1 × 20.0 mL) and dried with Na_2_SO_4_. Compound **9** was purified and isolated using silica-gel flash column chromatography (hexanes) as a white crystalline solid (80.0 mg, 40%); mp: 90–93 °C. ^1^H NMR (500 MHz, CDCl_3_) δ: 7.87–7.76 (m, 2H), 7.38 (dd, *J* = 7.7, 1.5 Hz, 1H), 7.35–7.26 (m, 2H), 7.24–7.17 (m, 3H), 5.23 (d, *J* = 1.7 Hz, 1H), 5.22 (d, *J* = 2.1 Hz, 1H), 4.19 (dd, *J* = 9.4, 2.3 Hz, 1H), 3.62 (d, *J* = 9.5 Hz, 1H), 2.28–2.18 (m, 2H), 2.12–2.04 (m, 1H), 1.86 (dp, *J* = 12.8, 2.8 Hz, 1H), 1.75 (p, *J* = 3.1 Hz, 1H), 1.68–1.58 (m, 5H), 1.53 (dt, *J* = 3.7, 1.8 Hz, 1H), 1.28 (dq, *J* = 12.9, 2.9 Hz, 1H), 1.14 (dp, *J* = 12.8, 2.8 Hz, 1H), 1.06 (dq, *J* = 12.8, 2.6 Hz, 1H). ^13^C­{^1^H} NMR (126 MHz, CDCl_3_) δ: 162.2, 137.3, 134.2, 133.4, 132.3, 131.7, 128.6, 127.9, 127.4, 127.2, 126.5, 123.6, 122.5, 107.1, 62.9, 47.1, 41.4, 40.6, 38.3, 34.8, 33.8, 33.7, 33.5, 33.1, 27.3, 27.1. FTIR: ν 2889, 2848, 1650, 1447 cm^–1^. HRMS (ESI-TOF) *m*/*z* [M]^+^ calcd. for C_26_H_26_ 338.2035, found 338.2017. UV/vis (acetonitrile, *c* = 3.50 × 10^–5^ M, 1 cm path length): λ_max_ = 220 nm (ε = 2.58 × 10^4^ L mol^–1^ cm^–1^), 275 nm (ε = 9.74 × 10^3^ L mol^–1^ cm^–1^).

### Photolysis of **5**

Precursor **5** (8.3 mg, 0.03 mmol) was dissolved in 0.75 mL of C_6_D_6_ in a quartz NMR tube, and *m*-xylene (5 μL, 4.3 mg, 0.04 mmol) was added as an internal standard. Progress of the photolysis was monitored every 15 min by ^1^H NMR until **5** had fully reacted (1 h). The yields of diphenylallene (**10**) and phenanthrene in the photolysate were determined to be 57% and 74% respectively. The identity of **10** was further confirmed by comparison to an authentic sample synthesized independently.^19^ Subsequently, the photolysate was analyzed by GC-MS, and the retention time and fragmentation pattern of the product **10** was found to be identical to that of the authentic sample. Attempts to isolate product **10** from precursor **5** using silica-gel flash column chromatography with hexanes as eluent were unsuccessful, as the compounds coeluted.

### Photolysis of **7**

Precursor **7** (3.8 mg, 0.013 mmol) was dissolved in 0.75 mL of C_6_D_6_ in a quartz NMR tube, and *m*-xylene (2.5 μL, 2.2 mg, 0.020 mmol) was added as an internal standard. Progress of the photolysis was monitored every 15 min by ^1^H NMR until **7** had fully reacted (45 min). The yields of phenylallene (**11**) and phenanthrene in the photolysate were determined to be 74% and 95% respectively. The identity of **11** was further confirmed by comparison to an authentic sample synthesized independently.^20,21^ Subsequently, the photolysate was analyzed by GC-MS, and the retention time and fragmentation pattern of the product **11** was found to be identical to that of the authentic sample. Allene **11** was isolated from the photolysis of 20 mg of precursor **7** (0.068 mmol) using silica-gel flash column chromatography with hexanes as eluent, affording **11** (2.3 mg, 29%) and phenanthrene (5.6 mg, 46%).

### Photolysis of **9**

Precursor **9** (3.4 mg, 0.01 mmol) was dissolved in 0.75 mL of C_6_D_6_ in a quartz NMR tube, and *m*-xylene (5 μL, 4.3 mg, 0.04 mmol) was added as an internal standard. Progress of the photolysis was monitored every 15 min by ^1^H NMR until **9** had fully reacted (45 min). The yields of adamantylideneallene (**12**) and phenanthrene in the photolysate were determined to be 80% and 96% respectively. The identity of **12** was further confirmed by comparison to an authentic sample synthesized independently.^22,23^ Subsequently, the photolysate was analyzed by GC-MS, and the retention time and fragmentation pattern of the product **12** was found to be identical to that of the authentic sample. After flash chromatography (silica gel/hexanes), allene **12** was isolated from the photolysis of 50 mg of precursor **9** (0.148 mmol), affording adamantylideneallene (5 mg, 21%) and phenanthrene (22 mg, 84%).

## Supplementary Material



## Data Availability

The data underlying this study are available in the published article and its Supporting Information.

## Notes

Experimental procedures and spectral data (PDF)
