# Supplementary material for: Photochemical Generation of Allenes from Phenanthrene-Based Methylenecyclobutanes
Source: J Org Chem. 2025 Jun 9;90(24):8429–33. doi: 10.1021/acs.joc.5c00781 (PMC12186522; doi:10.1021/acs.joc.5c00781)
Supplement: Supplementary file 1 [file jo5c00781_si_001.pdf]

ELECTRONIC SUPPORTING INFORMATION

Photochemical Generation of Allenes from Phenanthrene-Based Methylenecyclobutanes

*Zhazira Koldasbay, Alexander D. Roth, and Dasan M. Thamattoor**

Department of Chemistry, Colby College, Waterville, ME 04901 USA

*dmthamat@colby.edu

Table of Contents

(1) Characterization data for 2-Methylene-1,1-diphenyl-1,2,2a,10b-tetrahydrocyclobuta[l]phenanthrene **(5)**

(a) ^{1}H NMR spectrum…… S2
(b) ^{13}C NMR spectrum…… S3
(c) FTIR spectrum…… S4
(d) UV/Vis spectrum…… S5
(e) Crystal structure and salient data…… S6

(2) Characterization data for 1-methylene-2-phenyl-1,2,2a,10b-tetrahydrocyclobuta[l]phenanthrene **(7)**

(a) ^{1}H NMR spectrum…… S7
(b) ^{13}C NMR spectrum…… S8
(c) FTIR spectrum…… S9
(d) UV/Vis spectrum…… S10
(e) Crystal structure and salient data…… S11

(3) Characterization data for (5r,7r)-2'-Methylene-2a',10b'-dihydro-2'H-spiro[adamantane-2,1'-cyclobuta[l]phenanthrene] **(9)**

(a) ^{1}H NMR spectrum…… S12
(b) ^{13}C NMR spectrum…… S13
(c) FTIR spectrum…… S14
(d) UV/Vis spectrum…… S15
(e) Crystal structure and salient data…… S16

(4) Photolysis Data

(a) Photolysis **5**, 0 hours, NMR…… S17
(b) Photolysis **5**, 1 hour, NMR…… S18
(c) Photolysis **5**, 1 hour, GC-MS…… S19
(d) ^{1}H NMR spectrum of **10**…… S20
(e) GC-MS of **10**…… S21
(f) Photolysis **7**, 0 hours, NMR …… S22
(g) Photolysis **7**, 45 min, NMR…… S23
(h) Photolysis **7**, 45 min, NMR…… S24
(i) GC-MS of **11**…… S25
(j) Photolysis **9**, 0 hours, NMR…… S26
(k) Photolysis **9**, 1 hour, NMR…… S27
(l) Photolysis **9**, 1 hour, GC-MS…… S28
(m) ^{1}H NMR spectrum of **12**…… S29
(n) GC-MS of **12**…… S30

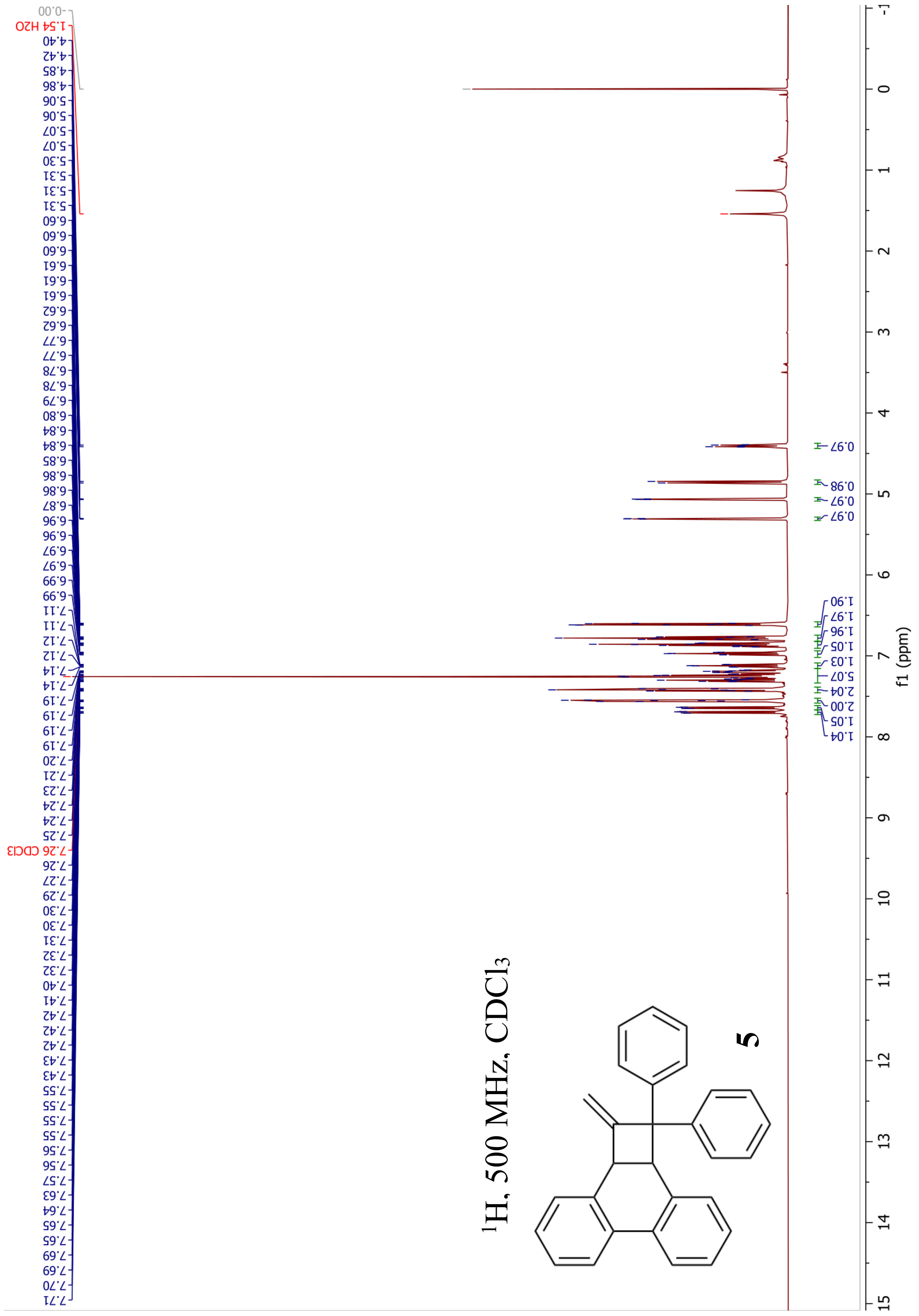

^{1}H, 500 MHz, $CDCl_3$
5
f1 (ppm)

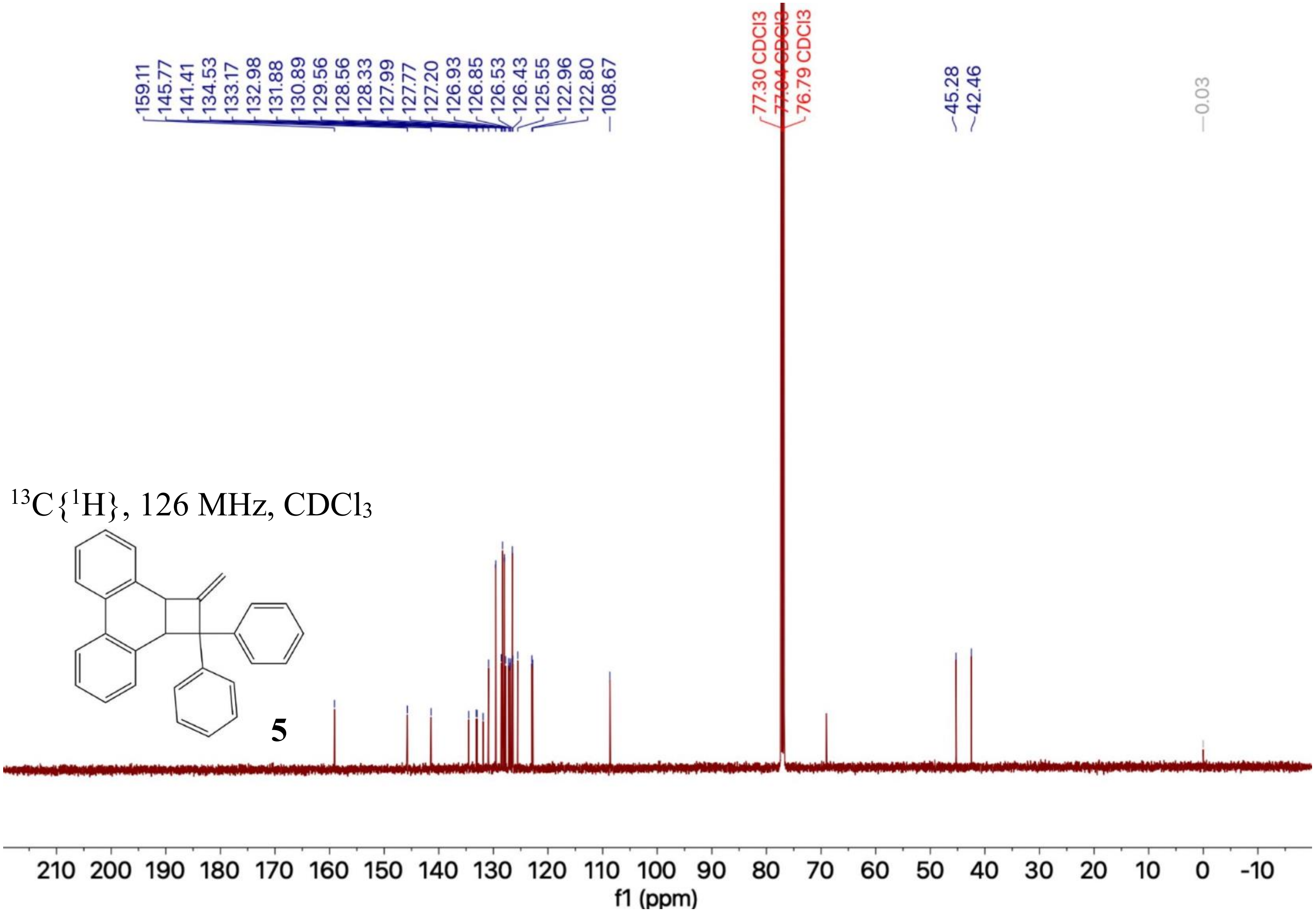

$^{13}C\{^{1}H\}$, 126 MHz, $CDCl_3$

5

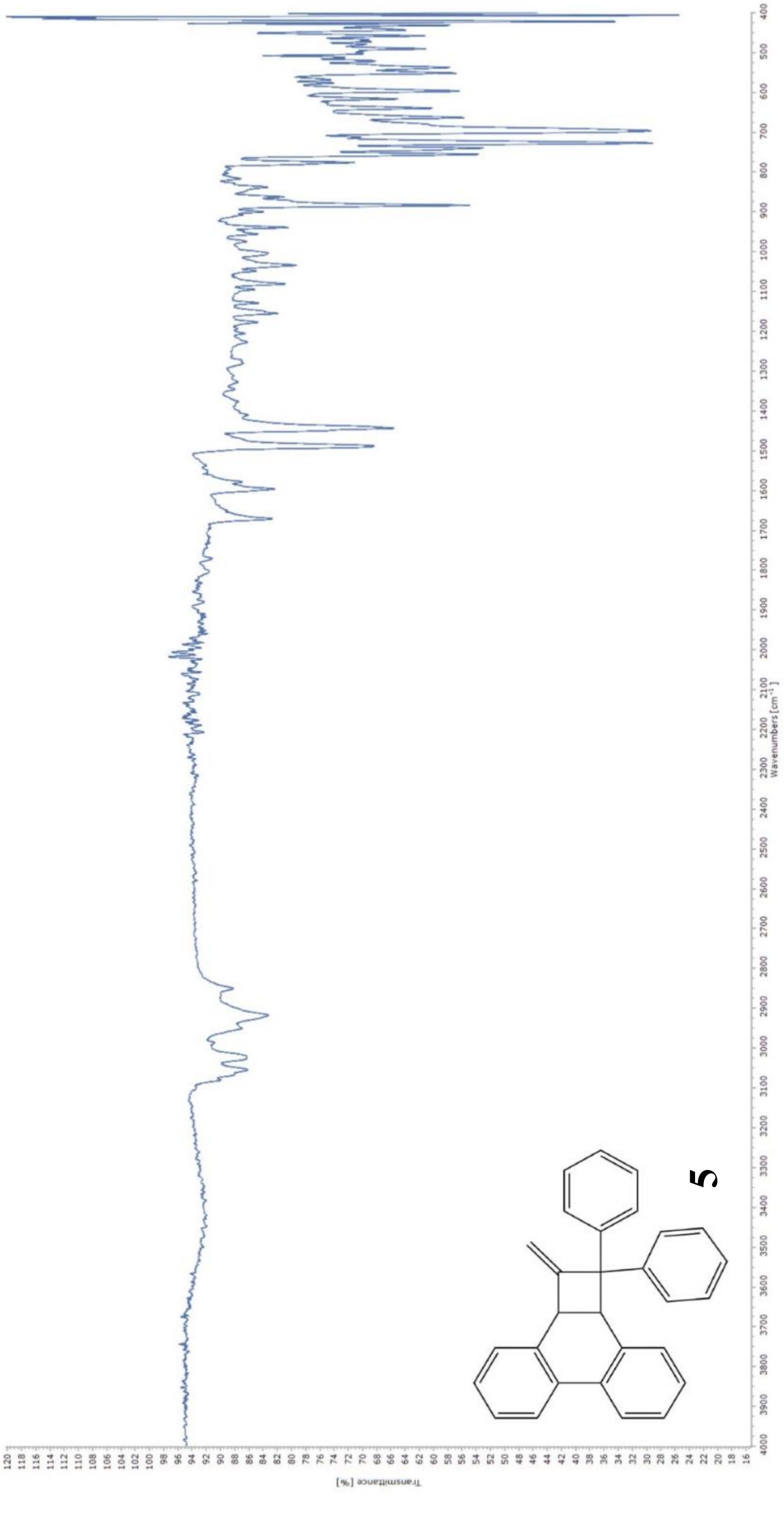
Transmittance [%]
Wavenumbers [cm-1]
5

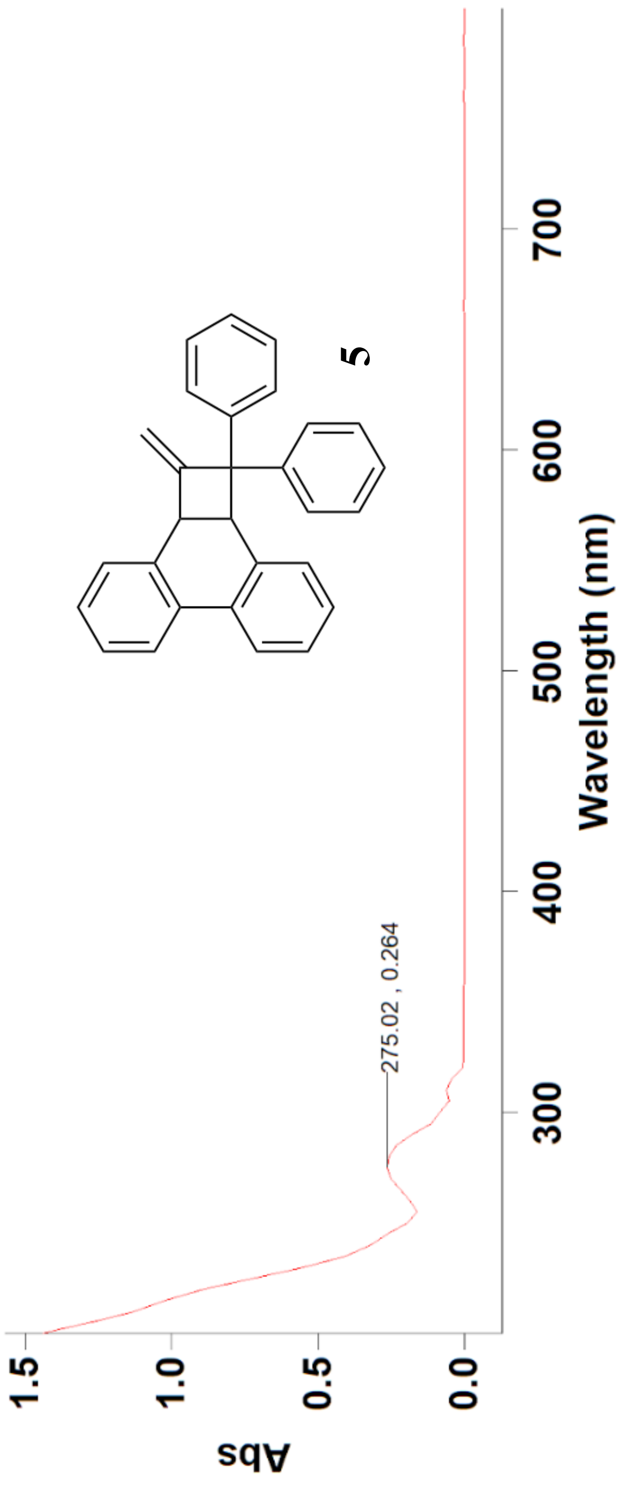
Abs
0.0
0.5
1.0
1.5
275.02 , 0.264
300
400
500
600
700
Wavelength (nm)
5

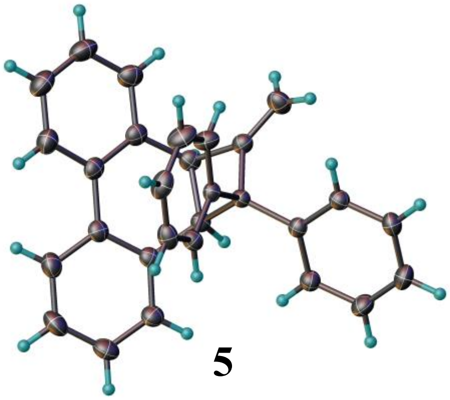

5

Empirical formula	$C_{29}H_{22}$
Formula weight	370.1722
Temperature/K	173.01
Crystal system	monoclinic
Space group	$P2_1/c$
a/Å	9.4493(3)
b/Å	22.3663(6)
c/Å	10.2756(3)
α/°	90
β/°	114.3160(10)
γ/°	90
Volume/Å^3	1979.05(10)
Z	4
ρ_{calc}g/cm^3	1.243
μ/mm^1	0.070
F(000)	784.0
Crystal size/mm^3	0.284 × 0.265 × 0.177
Radiation	Mo Kα (λ = 0.71073)
2Θ range for data collection/°	4.716 to 55.12
Index ranges	-12 ≤ h ≤ 12, -29 ≤ k ≤ 29, -13 ≤ l ≤ 13
Reflections collected	44404
Independent reflections	4564 [Rint = 0.0310, Rsigma = 0.0169]
Data/restraints/parameters	4564/0/262
Goodness-of-fit on F^2	1.034
Final R indexes [I>=2σ (I)]	R1 = 0.0421, wR2 = 0.1000
Final R indexes [all data]	R1 = 0.0508, wR2 = 0.1079
Largest diff. peak/hole / e Å^{-3}	0.26/-0.20
CCDC Number	2305847

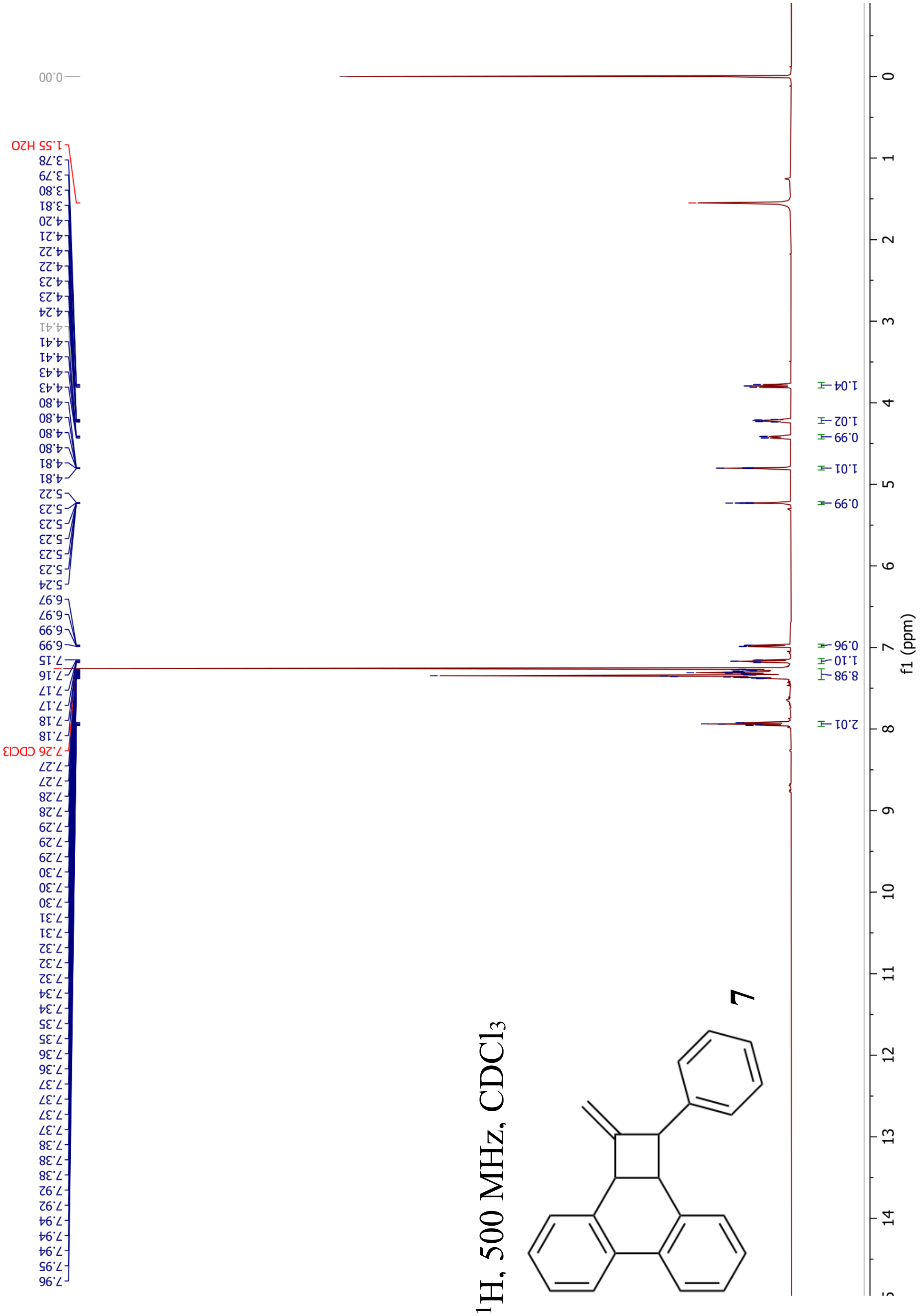

1H, 500 MHz, CDCl3
7
f1 (ppm)
1.55 H2O
7.26 CDCl3
0.00
1.04
1.02
0.99
1.01
0.99
0.96
1.10
8.98
2.01

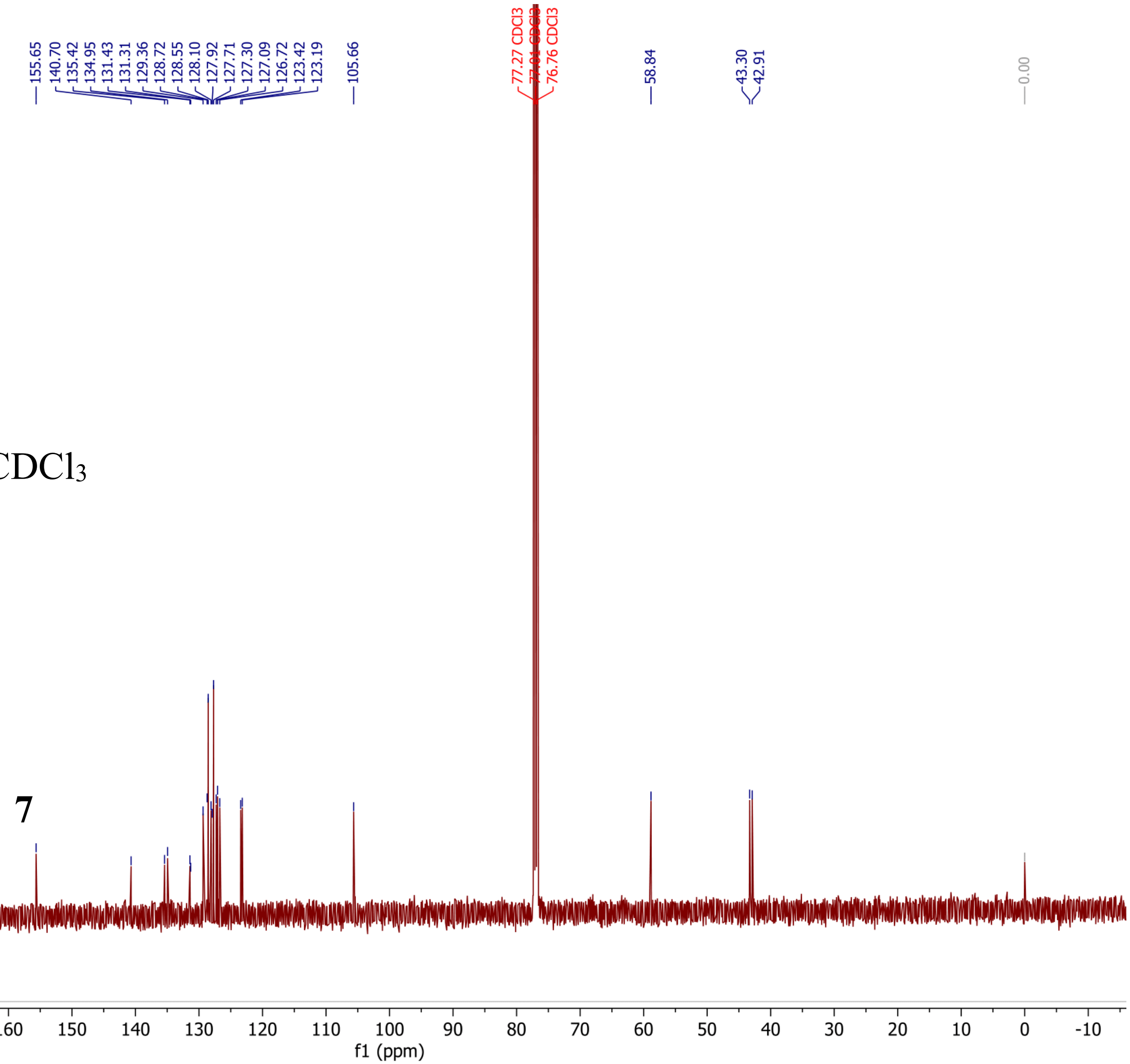

$^{13}C\{^{1}H\}$, 126 MHz, $CDCl_3$

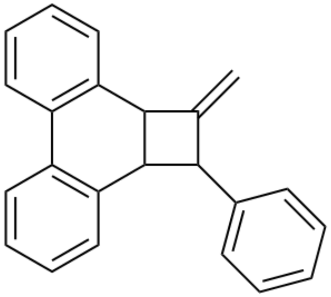

7

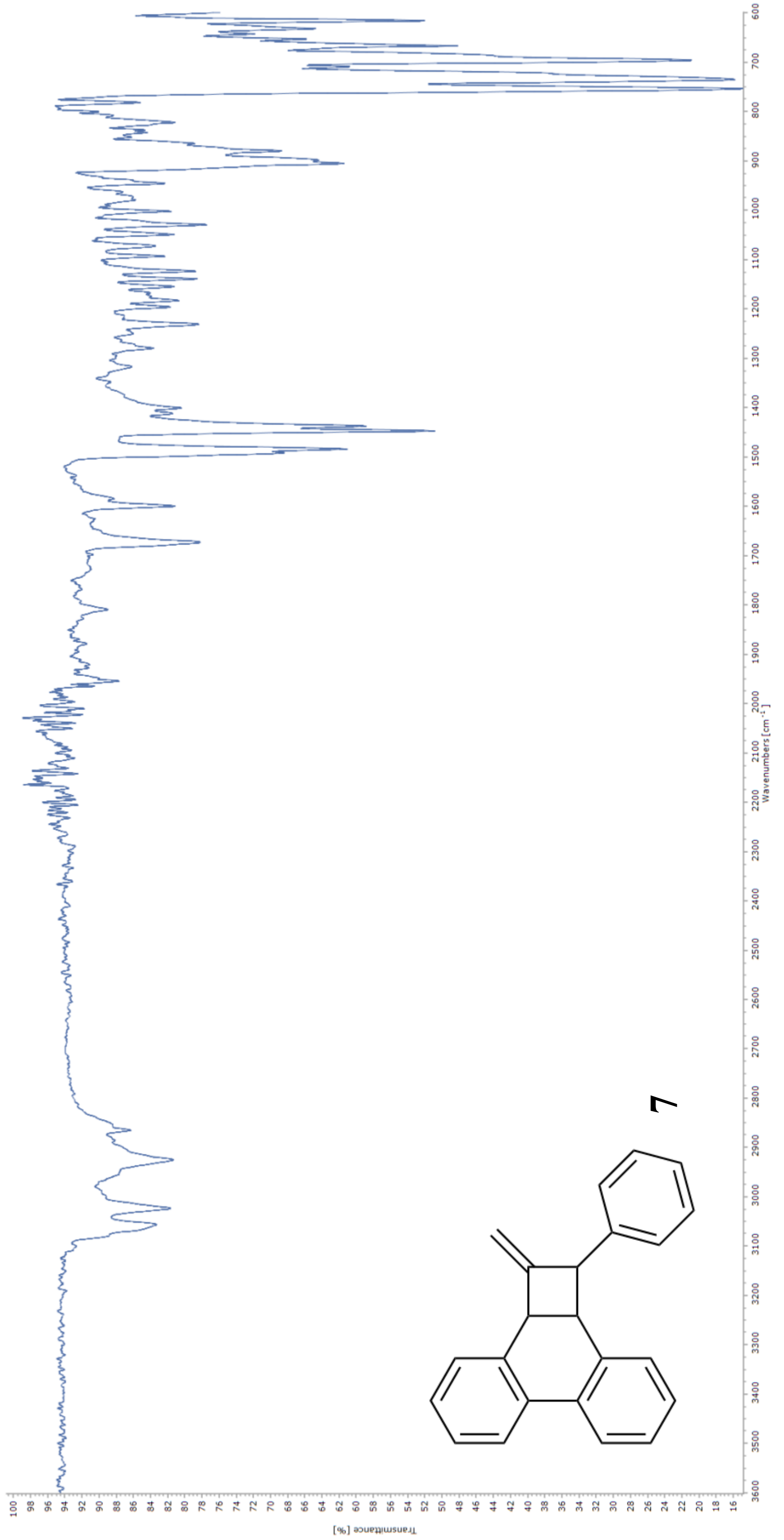
7
Transmittance [%]
Wavenumbers [cm⁻¹]

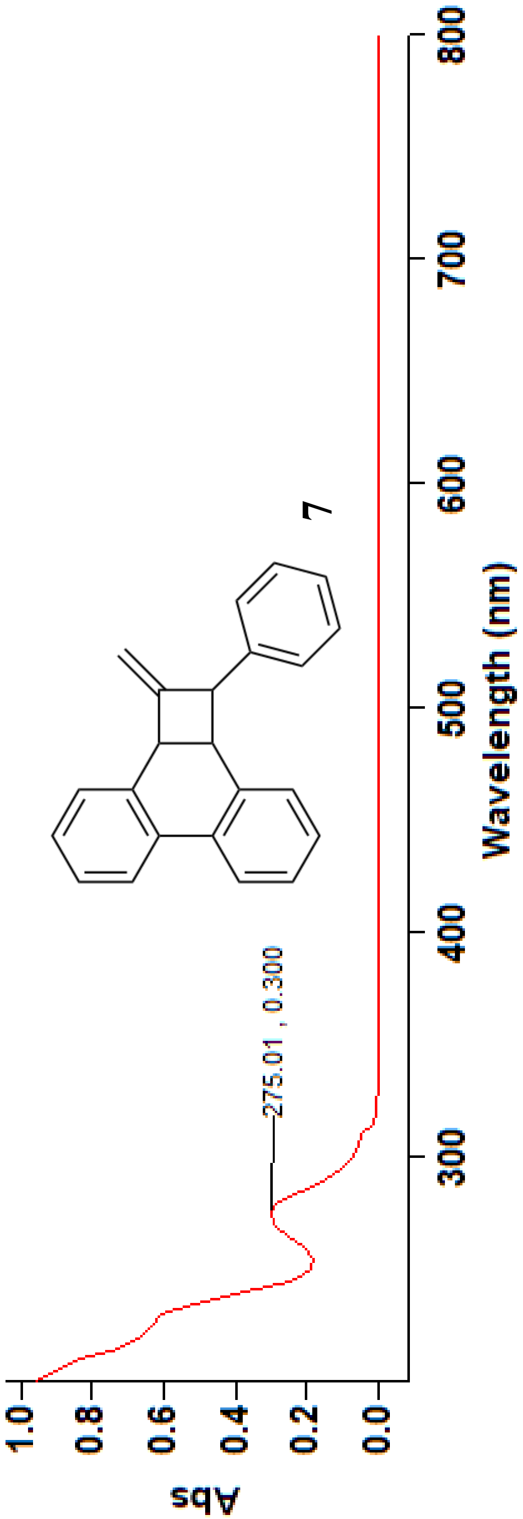
Abs
1.0
0.8
0.6
0.4
0.2
0.0
300
400
500
600
700
800
Wavelength (nm)
275.01 , 0.300
7

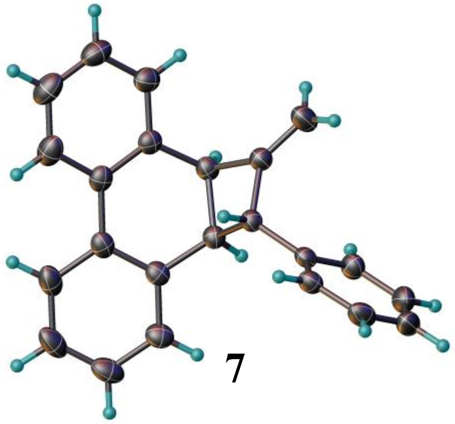

7

Empirical formula	$C_{23}H_{18}$
Formula weight	294.1409
Temperature/K	173.01
Crystal system	monoclinic
Space group	$P2_1/c$
a/Å	9.9327(2)
b/Å	17.4091(4)
c/Å	9.4918(2)
α/°	90
β/°	91.0990(10)
γ/°	90
Volume/$Å^3$	1641.01(6)
Z	4
$ρ_{calc}g/cm^3$	1.192
μ/mm^1	0.067
F(000)	624.0
Crystal size/mm^3	0.372 × 0.264 × 0.174
Radiation	Mo Kα (λ = 0.71073)
2Θ range for data collection/°	6.224 to 55.006
Index ranges	-12 ≤ h ≤ 12, -22 ≤ k ≤ 22, -12≤ l ≤ 12
Reflections collected	36976
Independent reflections	3756 [Rint = 0.0278, Rsigma = 0.0137]
Data/restraints/parameters	3756/0/208
Goodness-of-fit on F^2	1.034
Final R indexes [I>=2σ (I)]	R1 = 0.0413, wR2 = 0.0978
Final R indexes [all data]	R1 = 0.0526, wR2 = 0.1081
Largest diff. peak/hole / e $Å^{-3}$	0.23/-0.20
CCDC Number	2328907

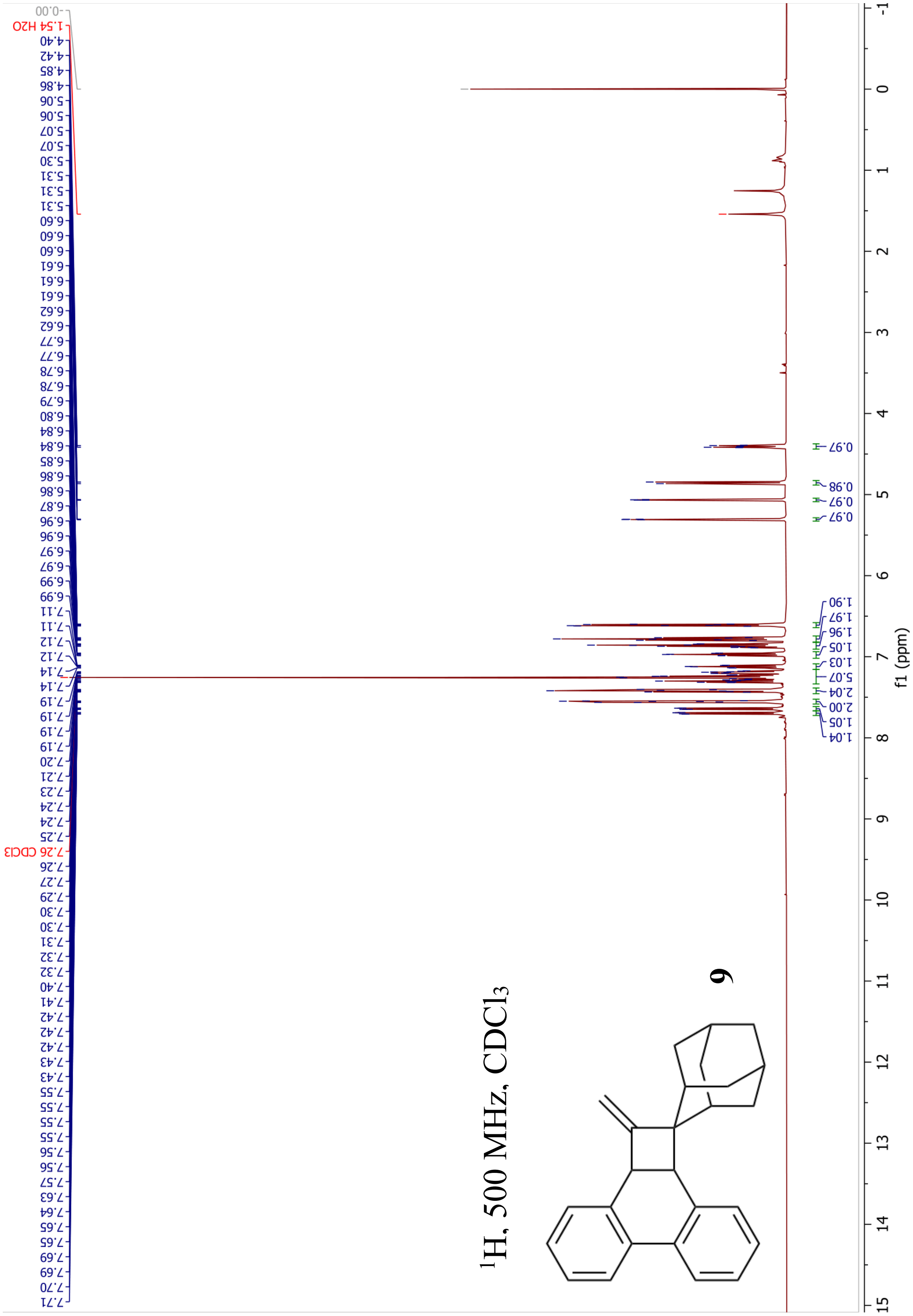

1H, 500 MHz, CDCl3
9
f1 (ppm)
-0.00
1.54 H2O
4.40
4.42
4.85
4.86
5.06
5.06
5.07
5.07
5.30
5.31
5.31
5.31
6.60
6.60
6.60
6.61
6.61
6.61
6.62
6.62
6.77
6.77
6.78
6.78
6.79
6.80
6.84
6.84
6.85
6.86
6.86
6.87
6.96
6.96
6.97
6.97
6.99
6.99
7.11
7.11
7.12
7.12
7.14
7.14
7.19
7.19
7.19
7.19
7.20
7.21
7.23
7.24
7.24
7.25
7.26 CDCl3
7.26
7.27
7.29
7.30
7.30
7.31
7.32
7.32
7.40
7.41
7.42
7.42
7.42
7.43
7.43
7.55
7.55
7.55
7.55
7.56
7.56
7.57
7.63
7.64
7.65
7.65
7.69
7.69
7.70
7.71
0.97
0.98
0.97
0.97
1.90
1.97
1.96
1.05
1.03
5.07
2.04
2.00
1.05
1.04
-1
0
1
2
3
4
5
6
7
8
9
10
11
12
13
14
15

$^{13}C\{^{1}H\}$, 126 MHz, $CDCl_3$

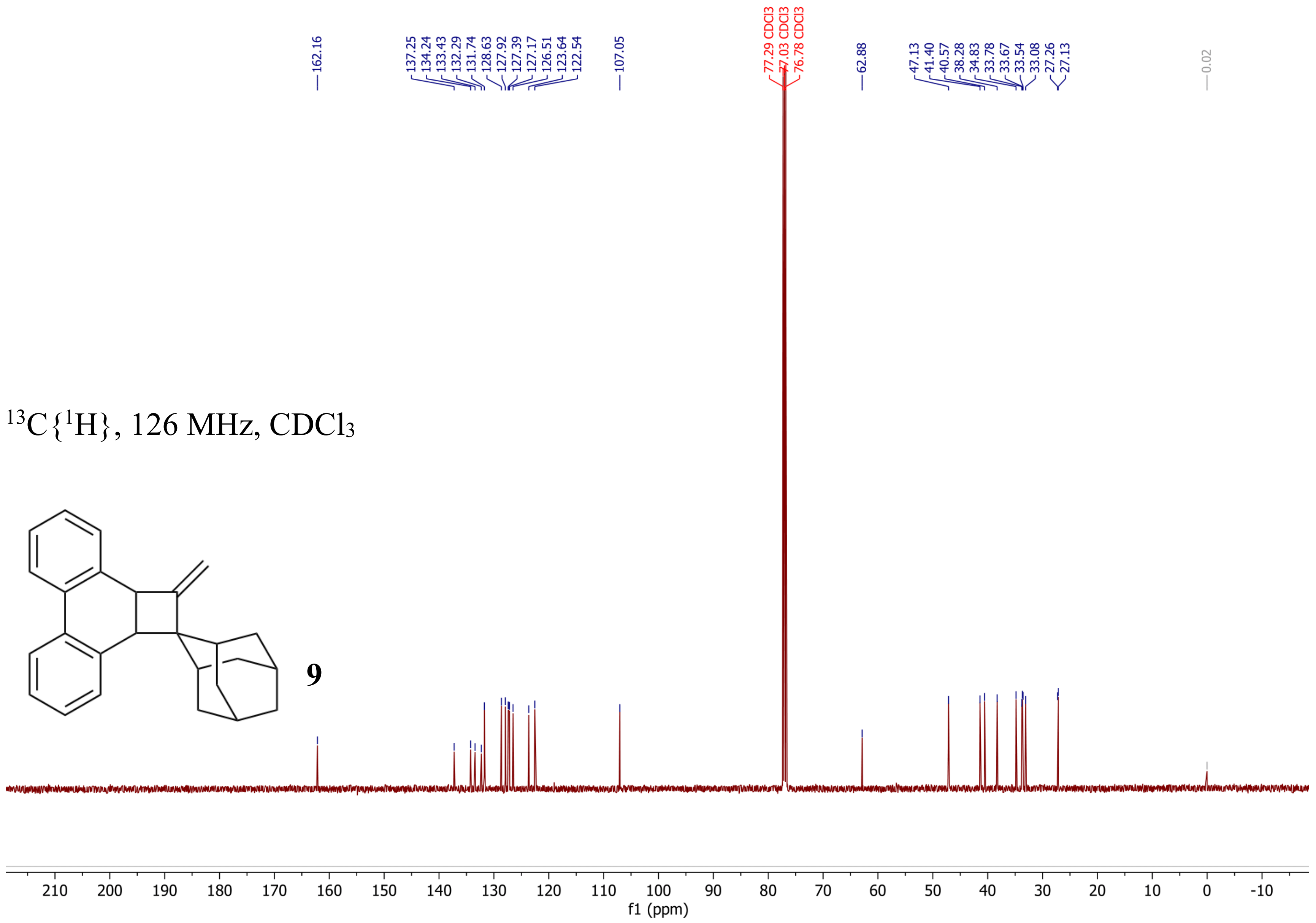

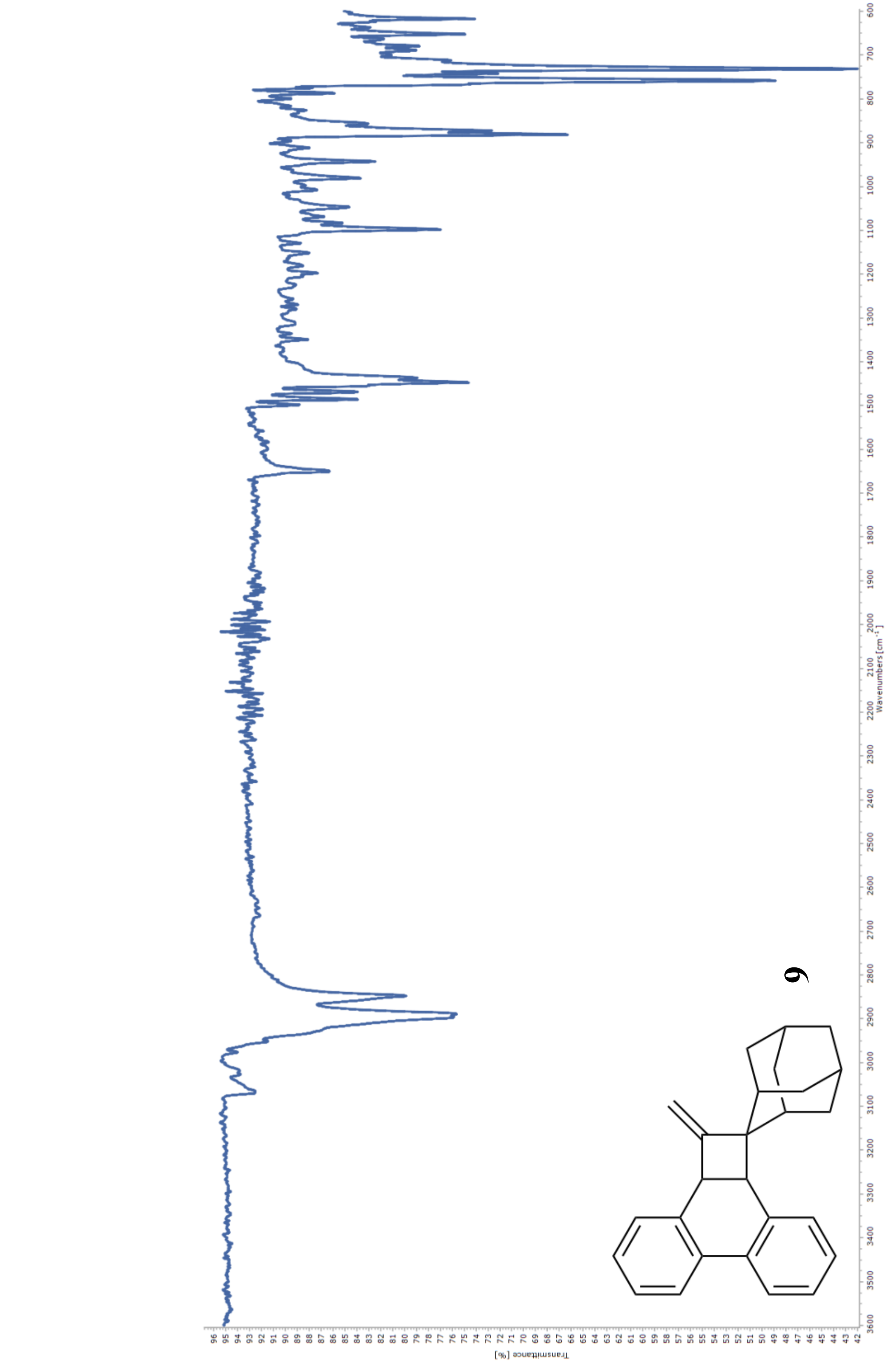
9
Transmittance [%]
Wavenumbers [cm⁻¹]

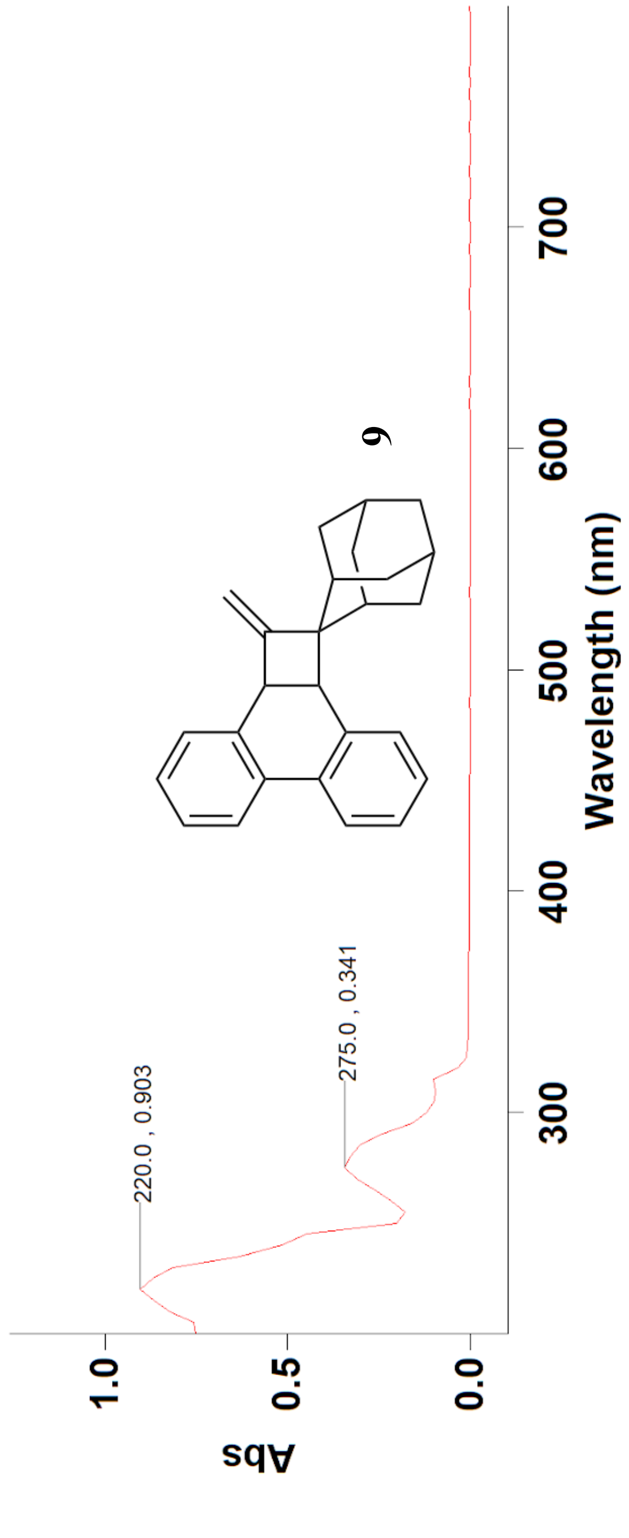
Abs
1.0
0.5
0.0
220.0 , 0.903
275.0 , 0.341
9
300
400
500
600
700
Wavelength (nm)

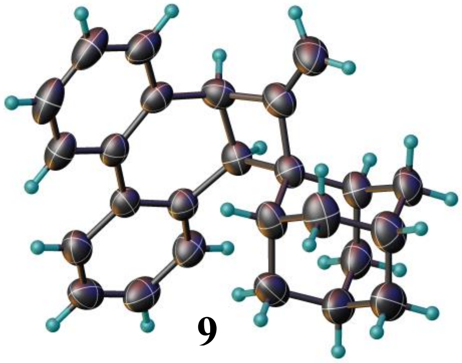

9

Empirical formula	$C_{26}H_{26}$
Formula weight	338.2035
Temperature/K	173.01
Crystal system	orthorhombic
Space group	Pbcn
a/Å	38.4858(11)
b/Å	9.1006(2)
c/Å	10.5166(3)
α/°	90
β/°	90
γ/°	90
Volume/Å^3	3683.38(17)
Z	8
ρ_{calc}g/cm^3	1.221
μ/mm^1	0.068
F(000)	1456.0
Crystal size/mm^3	0.229 × 0.194 × 0.043
Radiation	Mo Kα (λ = 0.71073)
2Θ range for data collection/°	5.488 to 50.7
Index ranges	-45 ≤ h ≤ 46, -10 ≤ k ≤ 10, -12 ≤ l ≤ 12
Reflections collected	46034
Independent reflections	3371 [Rint = 0.0755, Rsigma = 0.0285]
Data/restraints/parameters	3371/0/236
Goodness-of-fit on F^2	1.099
Final R indexes [I>=2σ (I)]	R1 = 0.0714, wR2 = 0.1637
Final R indexes [all data]	R1 = 0.1235, wR2 = 0.2104
Largest diff. peak/hole / e Å^{-3}	0.22/-0.21
CCDC Number	2430114

Photolysis of **5** (t = 0 hours)

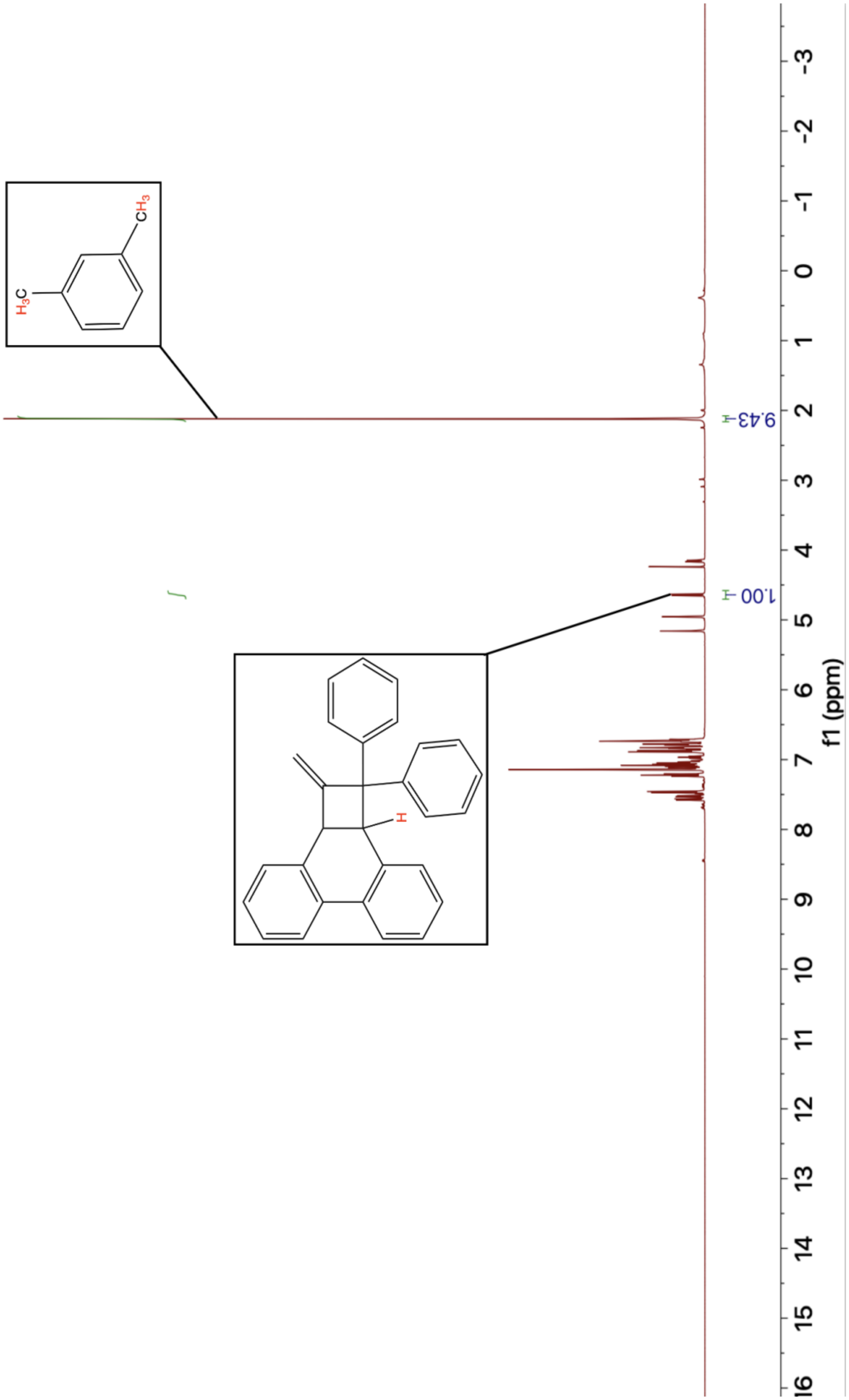

Photolysis of **5** (t = 1 hour)

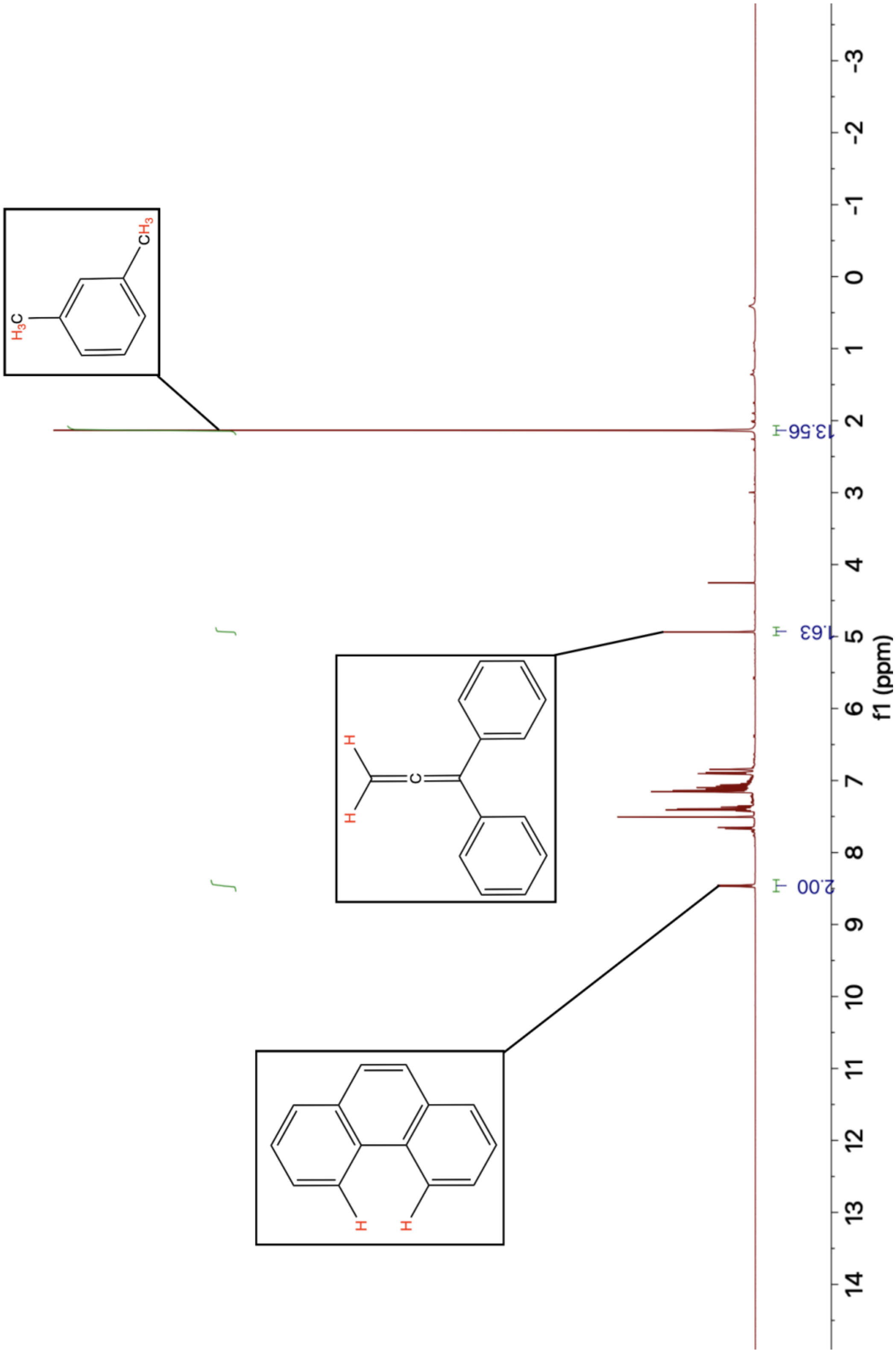

Photolysis of **5** (t = 1 hour)

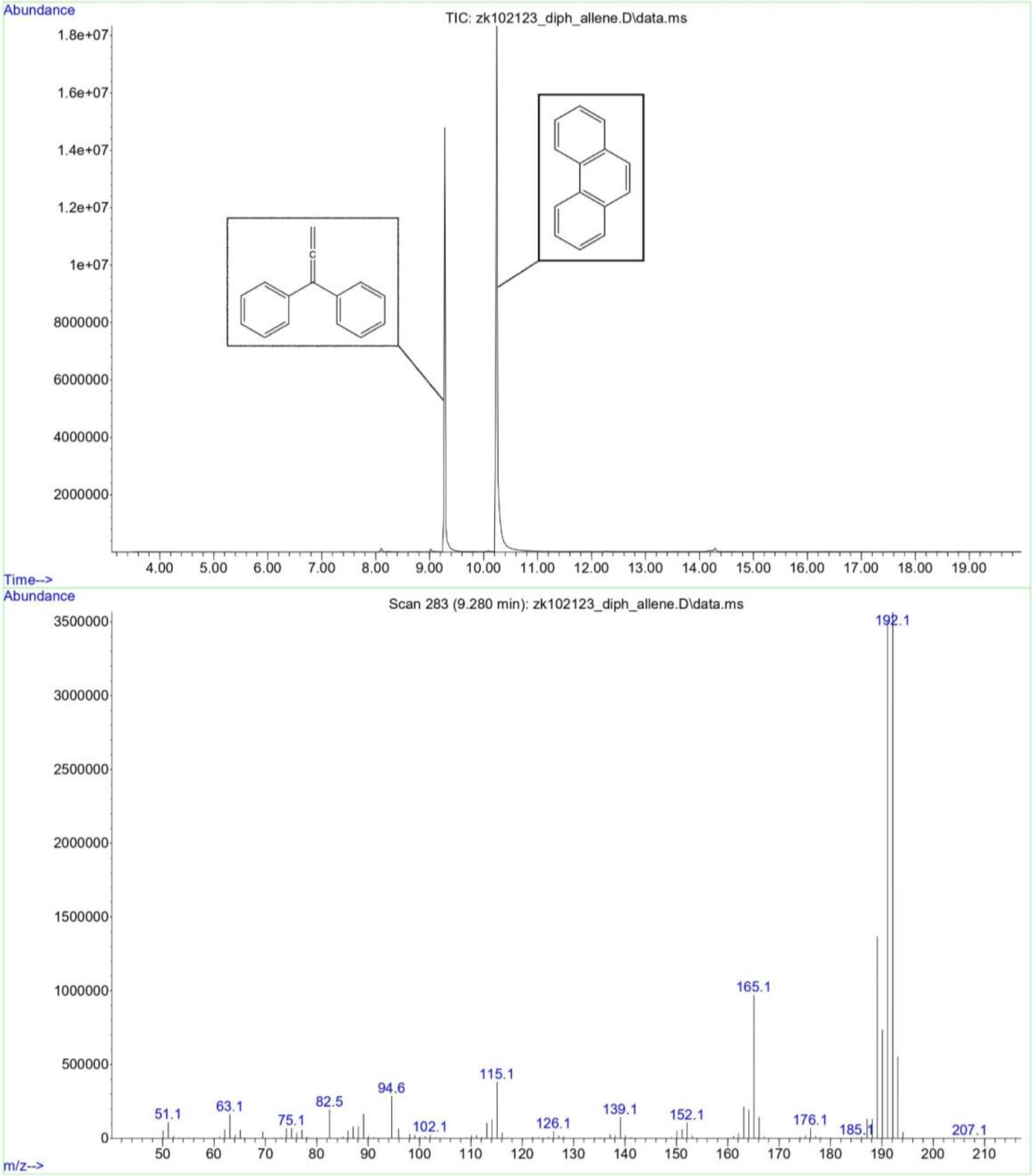

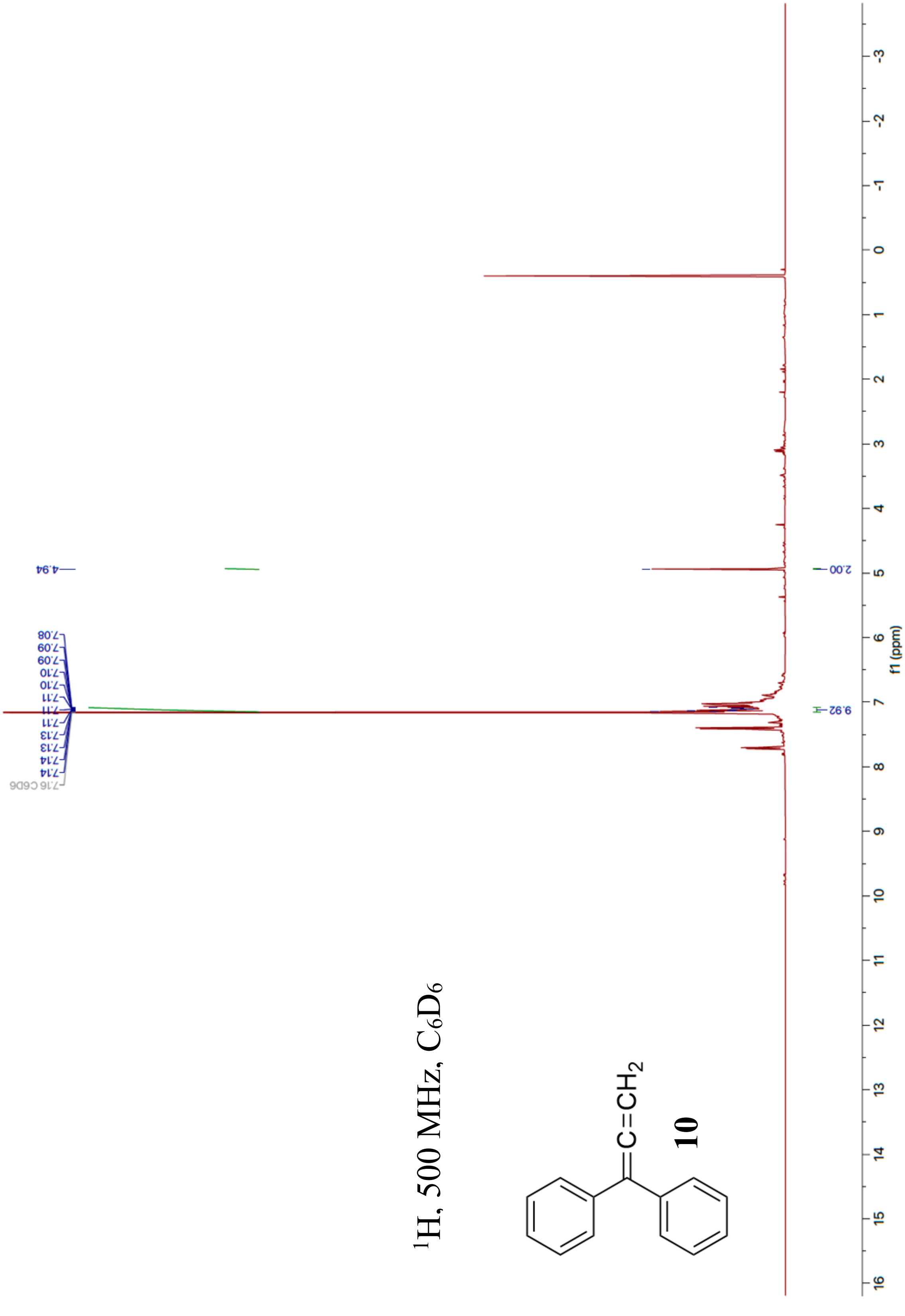

1H, 500 MHz, C6D6
C=CH2
10
f1 (ppm)
4.94
2.00
9.92
7.16 C6D6

\

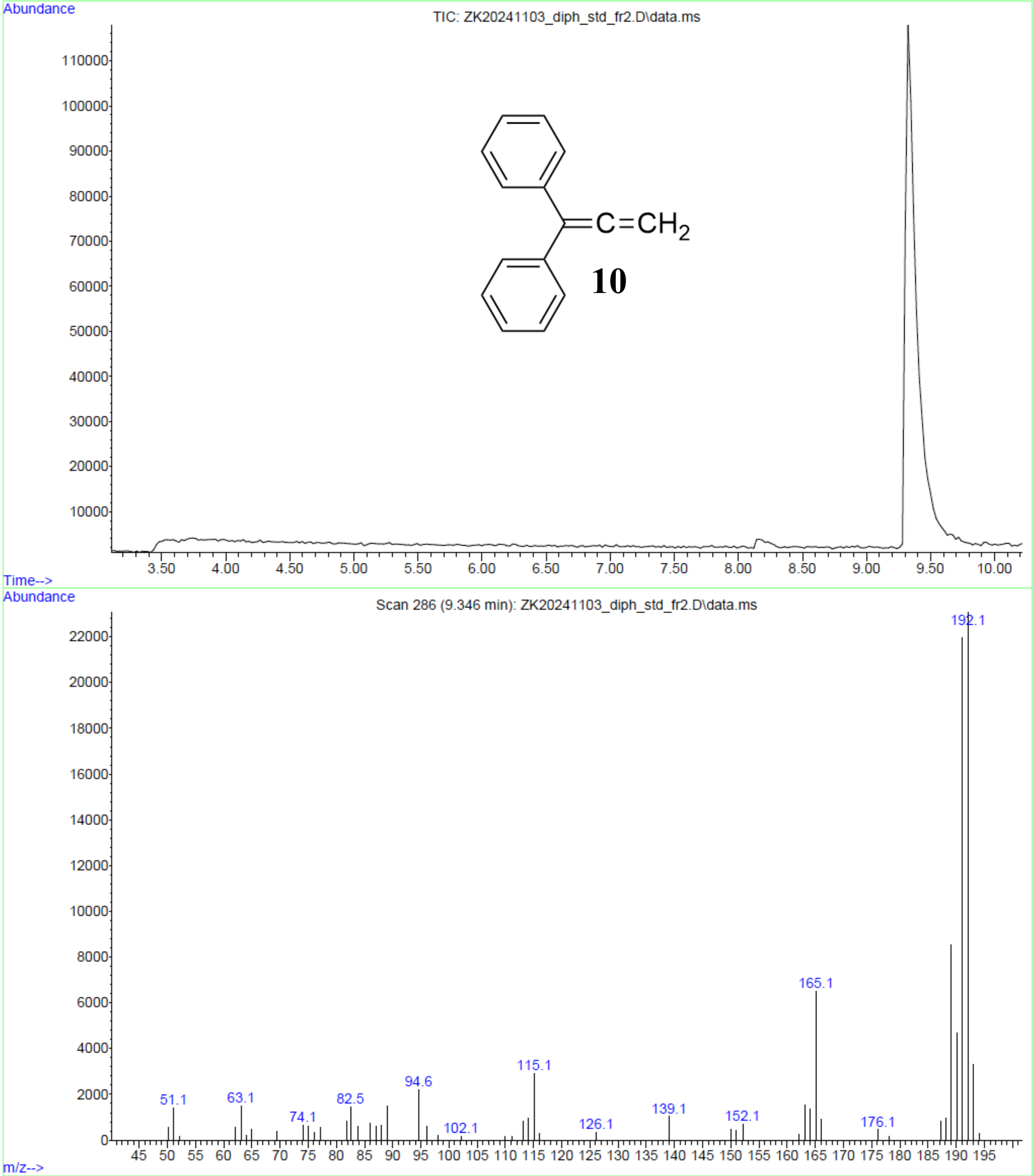
Abundance
TIC: ZK20241103_diph_std_fr2.D\data.ms
C=CH2
10
Time-->
Abundance
Scan 286 (9.346 min): ZK20241103_diph_std_fr2.D\data.ms
192.1
165.1
115.1
94.6
82.5
74.1
63.1
51.1
102.1
126.1
139.1
152.1
176.1
m/z-->

Photolysis of **7** (t = 0 hours)

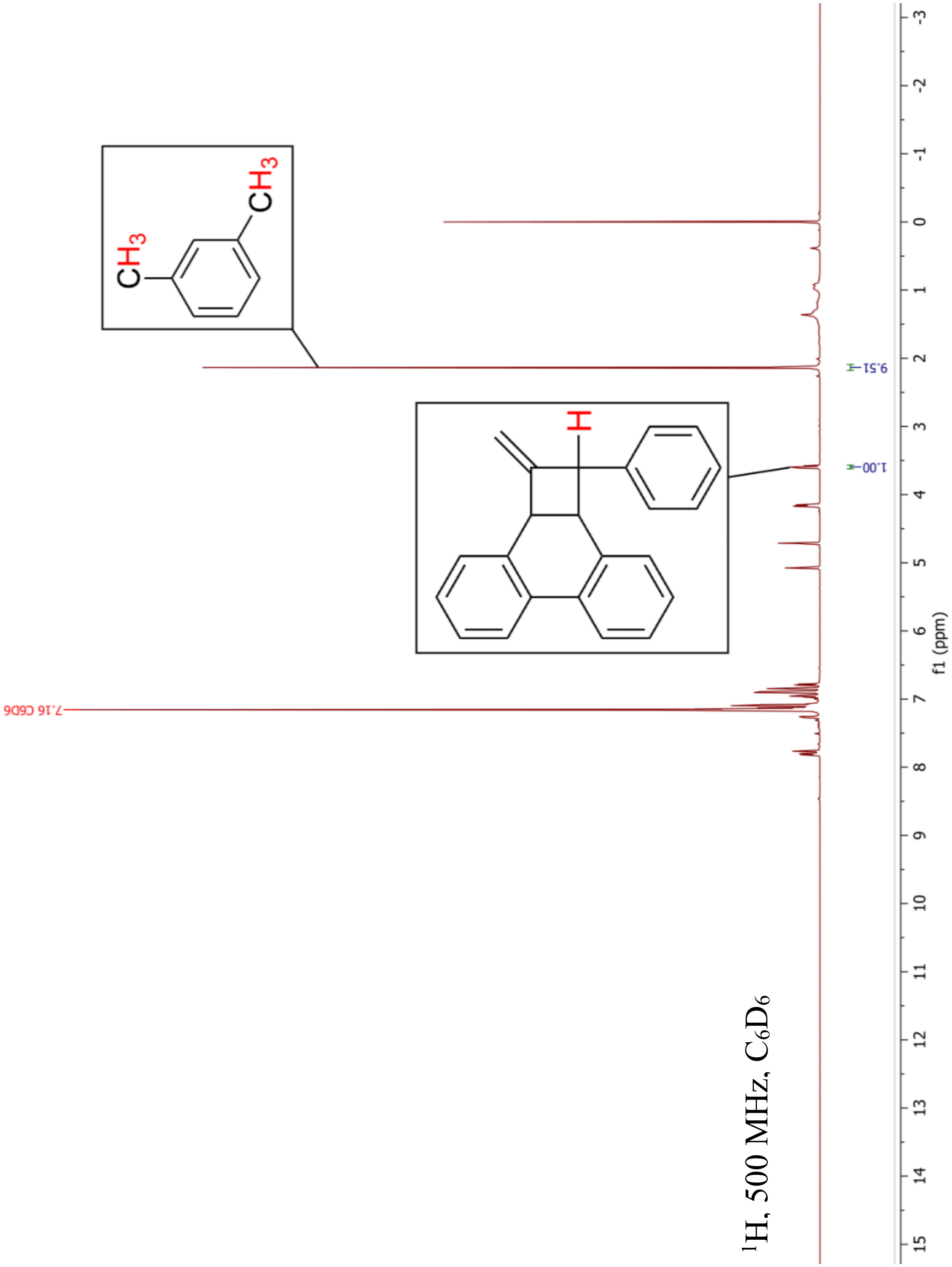

Photolysis of **7** (t = 45 min)

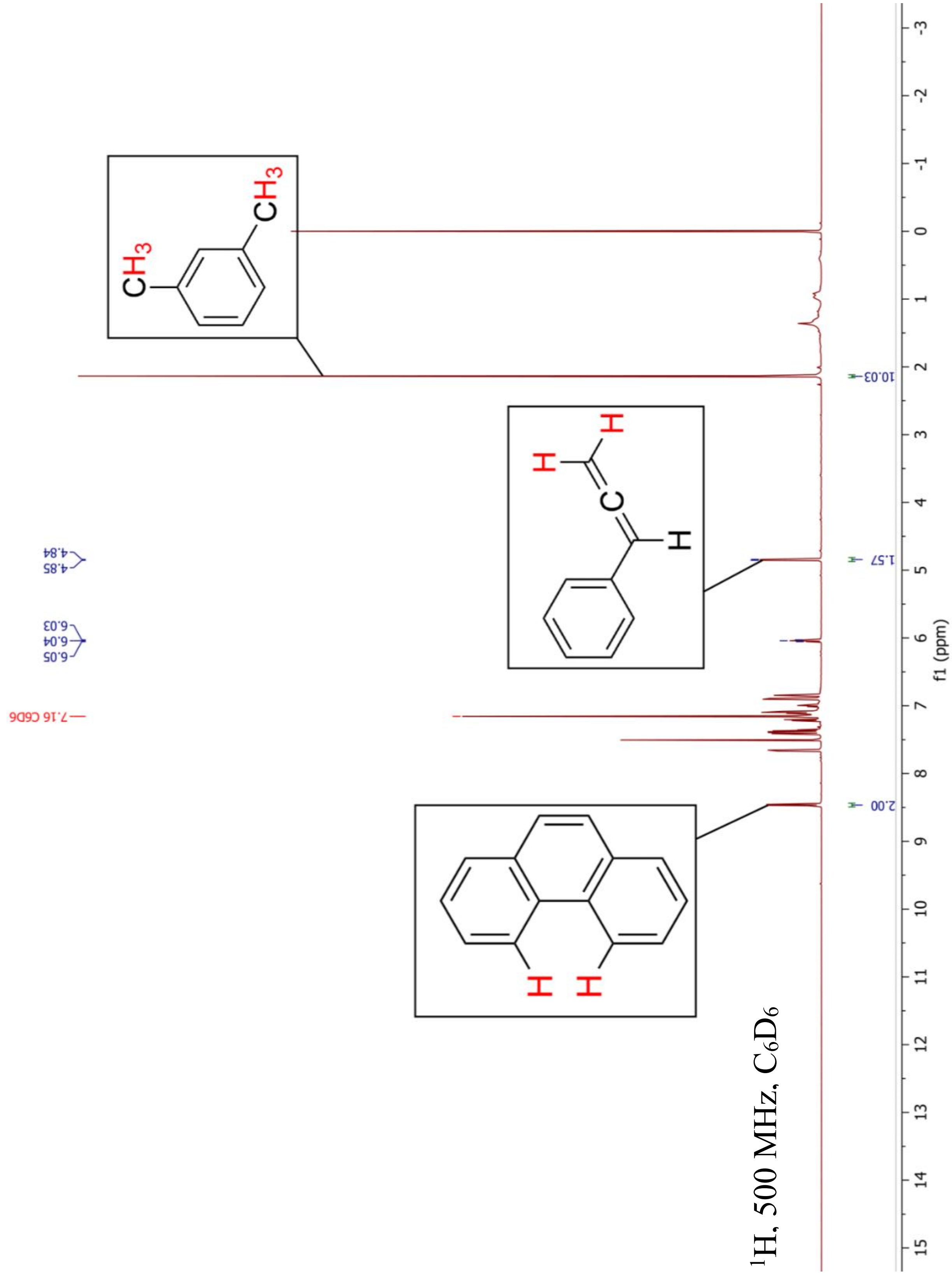

Photolysis of **7** (t = 45 min)

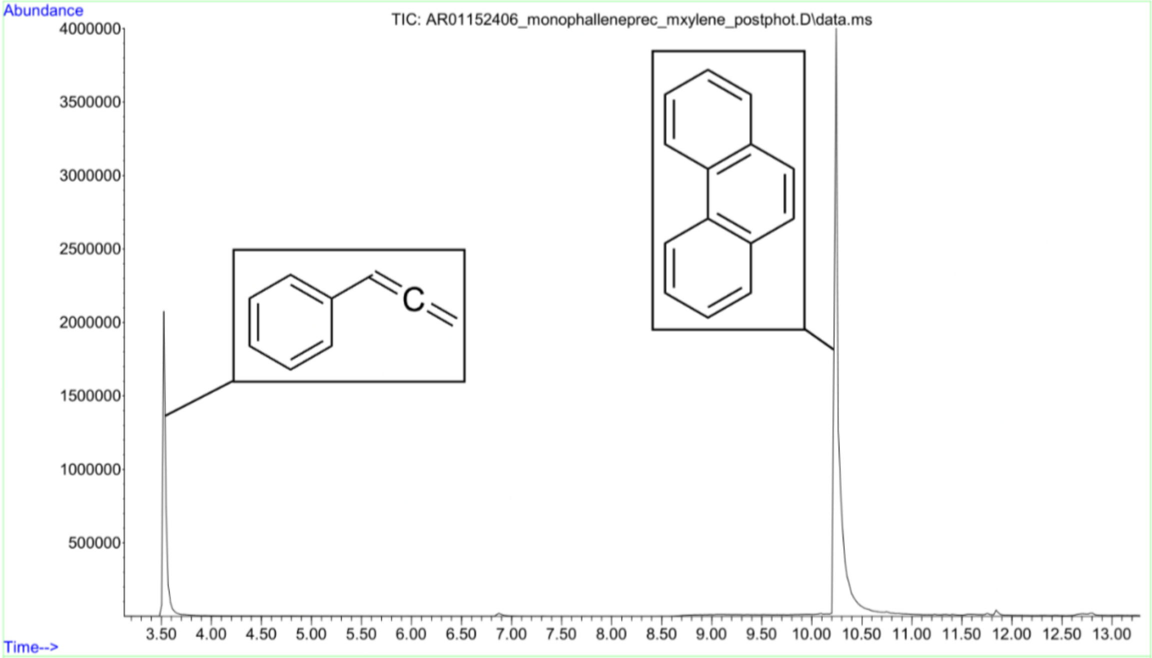

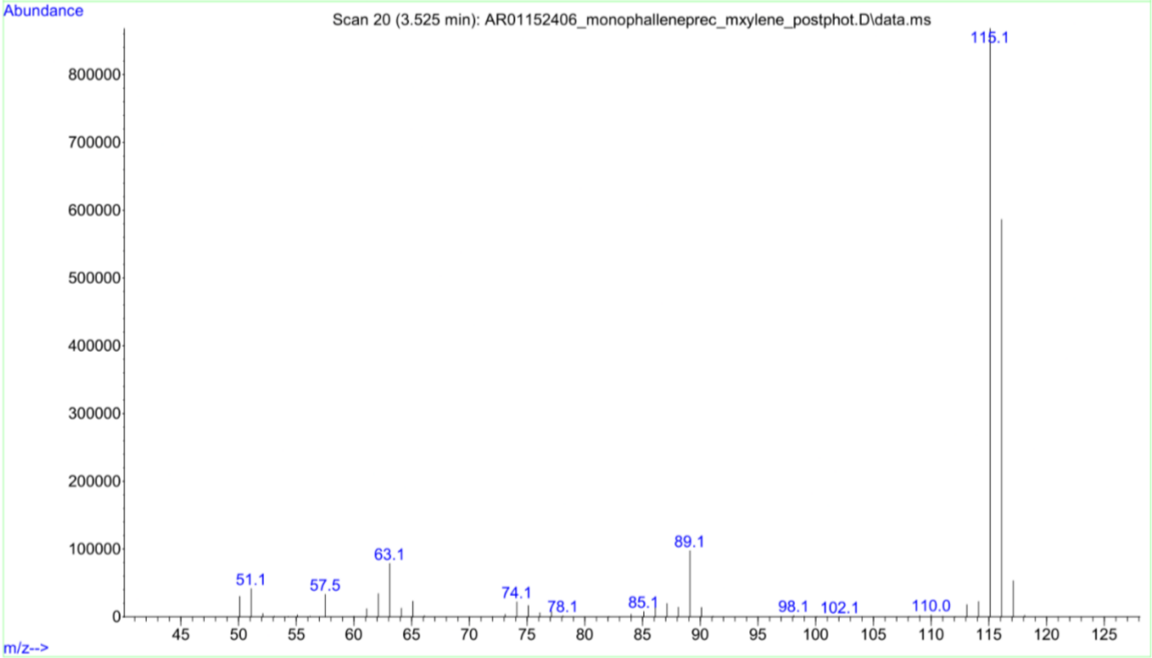

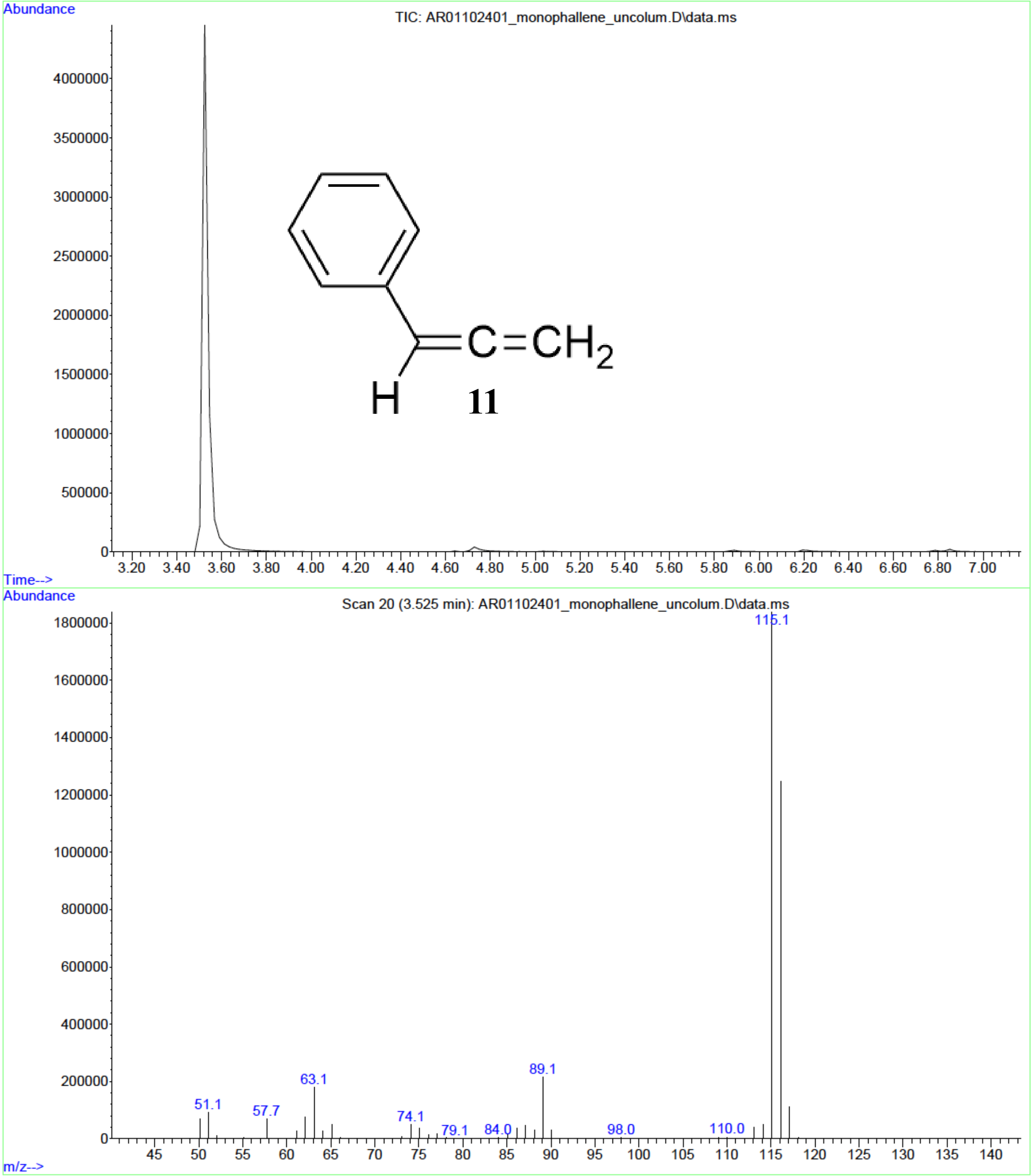
Abundance
TIC: AR01102401_monophallene_uncolum.D\data.ms
4000000
3500000
3000000
2500000
2000000
1500000
1000000
500000
0
3.20 3.40 3.60 3.80 4.00 4.20 4.40 4.60 4.80 5.00 5.20 5.40 5.60 5.80 6.00 6.20 6.40 6.60 6.80 7.00
Time-->
C=CH2
H
11
Abundance
Scan 20 (3.525 min): AR01102401_monophallene_uncolum.D\data.ms
1800000
1600000
1400000
1200000
1000000
800000
600000
400000
200000
0
115.1
89.1
63.1
51.1
57.7
74.1
79.1
84.0
98.0
110.0
45 50 55 60 65 70 75 80 85 90 95 100 105 110 115 120 125 130 135 140
m/z-->

Photolysis of **9** (t = 0 hours)

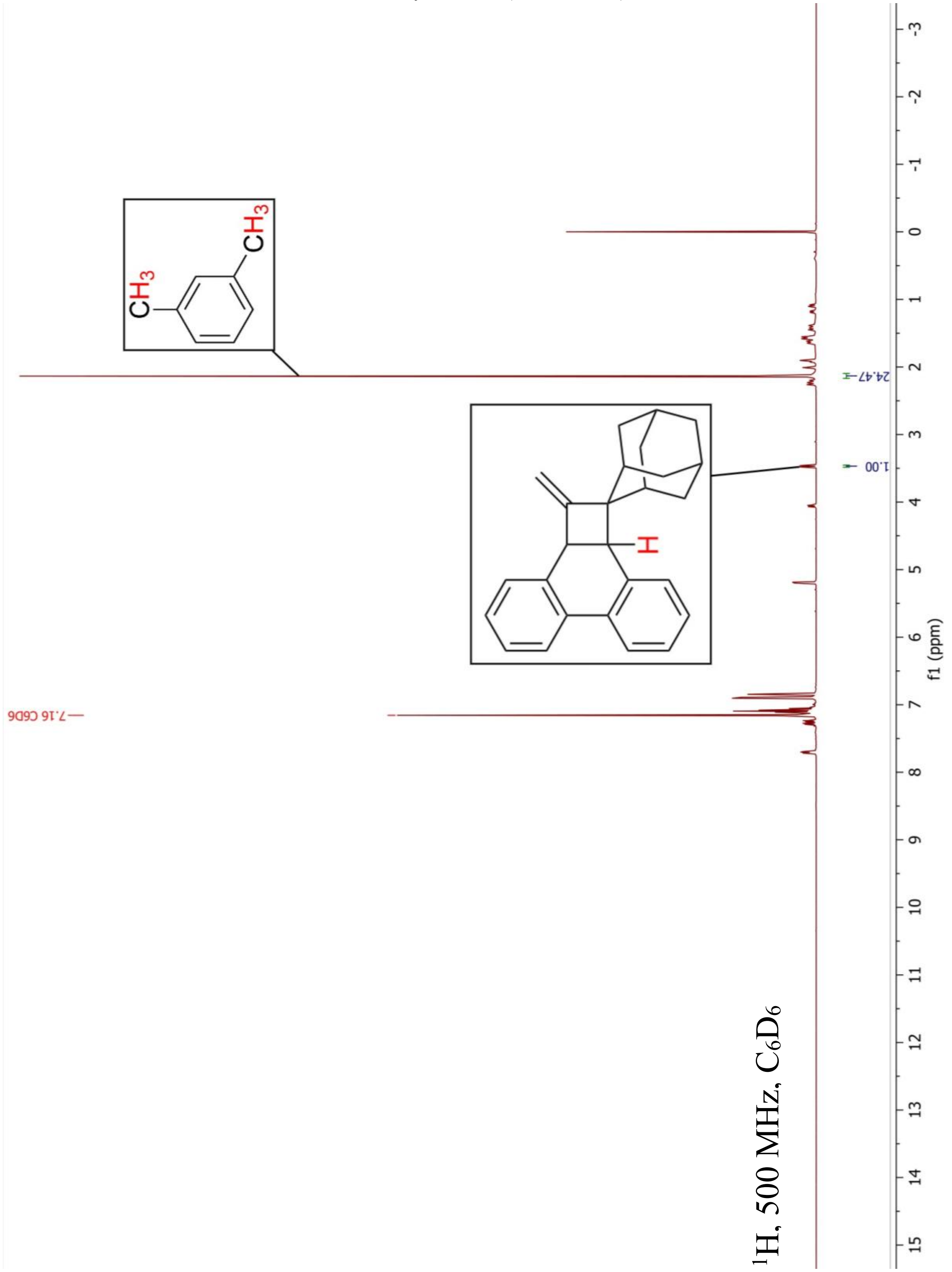

Photolysis of **9** (t = 1 hour)

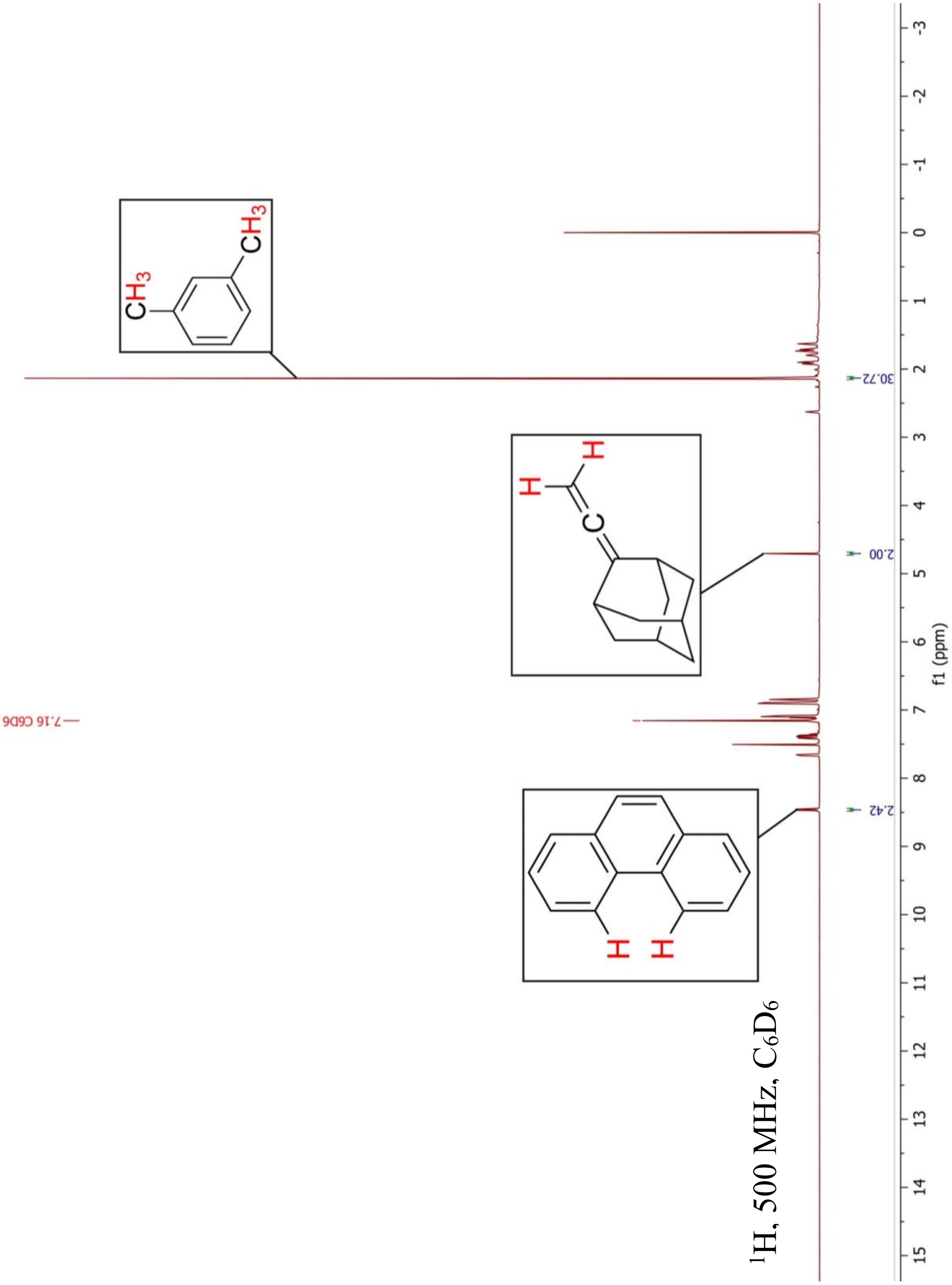

Photolysis of **9** (t = 1 hour)

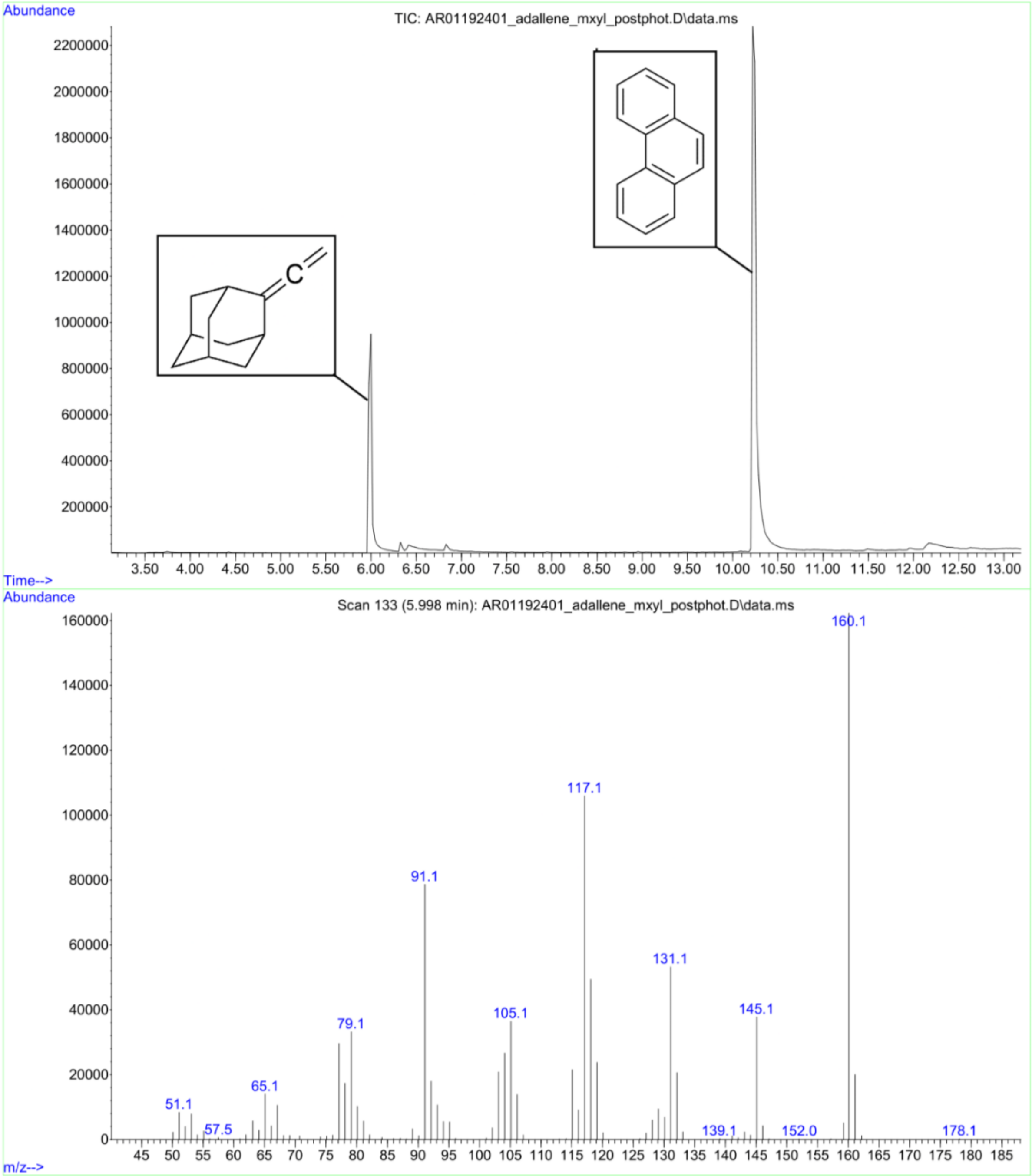

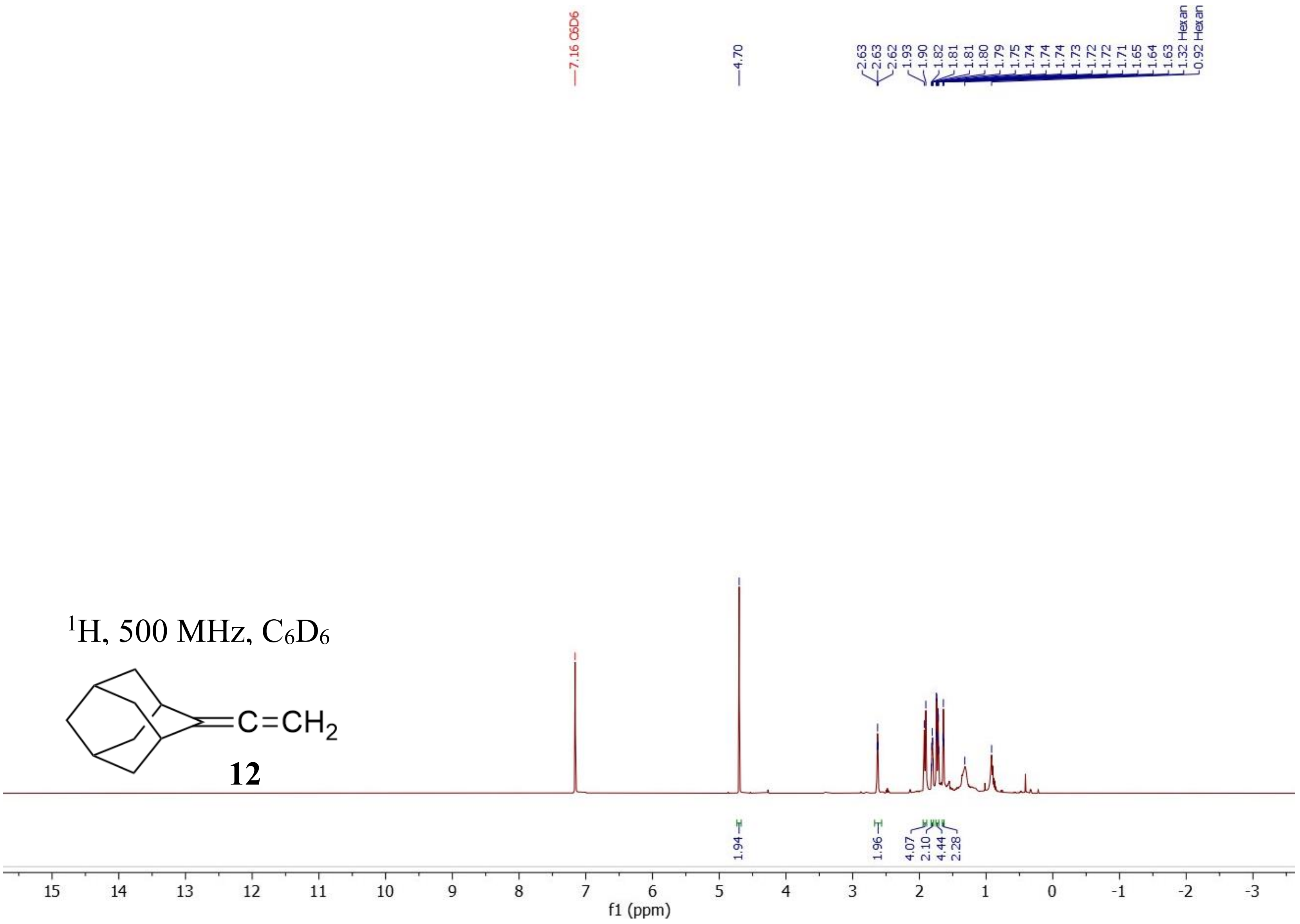

^1H, 500 MHz, C_6D_6
C=CH2
12
7.16 C6D6
4.70
2.63
2.63
2.62
1.93
1.90
1.82
1.81
1.81
1.80
1.79
1.75
1.74
1.74
1.74
1.73
1.72
1.72
1.71
1.65
1.64
1.63
1.32 Hexan
0.92 Hexan
1.94
1.96
4.07
2.10
4.44
2.28
15 14 13 12 11 10 9 8 7 6 5 4 3 2 1 0 -1 -2 -3
f1 (ppm)

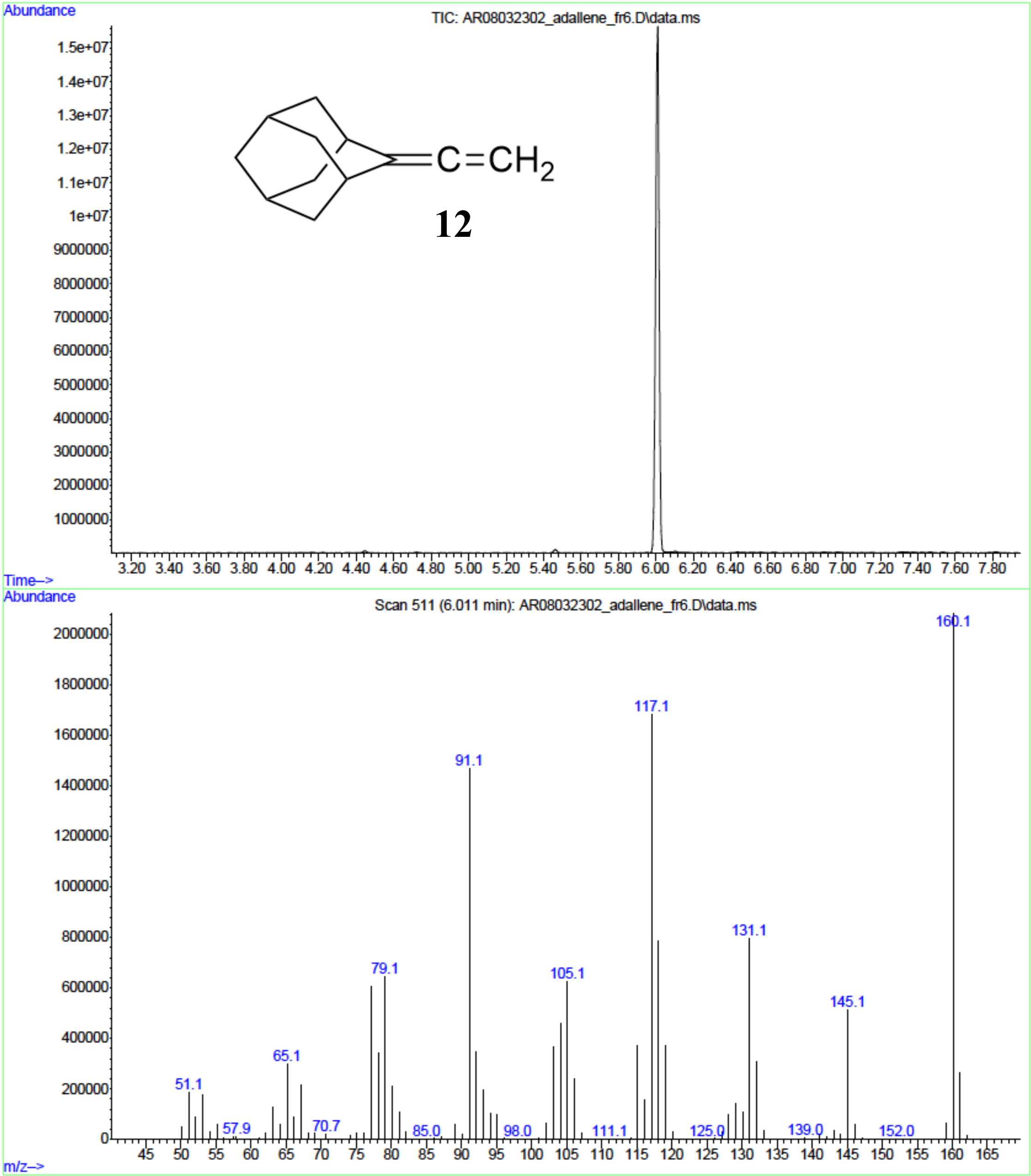
Abundance
TIC: AR08032302_adallene_fr6.D\data.ms
C=CH2
12
Time-->
Abundance
Scan 511 (6.011 min): AR08032302_adallene_fr6.D\data.ms
160.1
117.1
91.1
131.1
79.1
105.1
145.1
65.1
51.1
57.9
70.7
85.0
98.0
111.1
125.0
139.0
152.0
m/z-->
